# Conic formulation of QPCCs applied to truly sparse QPs

**DOI:** 10.1007/s10589-022-00440-5

**Published:** 2022-12-13

**Authors:** Immanuel M. Bomze, Bo Peng

**Affiliations:** 1 VCOR and VGSCO, University of Vienna, Oskar-Morgenstern-Platz 1, 1090 Vienna, Austria; 2 VGSCO, University of Vienna, Oskar-Morgenstern-Platz 1, 1090 Vienna, Austria

**Keywords:** Copositive optimization, Conic relaxation, Quadratic optimization, Sparsity, Complementarity constraints

## Abstract

We study (nonconvex) quadratic optimization problems with complementarity constraints, establishing an exact completely positive reformulation under—apparently new—mild conditions involving only the constraints, not the objective. Moreover, we also give the conditions for strong conic duality between the obtained completely positive problem and its dual. Our approach is based on purely continuous models which avoid any branching or use of large constants in implementation. An application to pursuing interpretable sparse solutions of quadratic optimization problems is shown to satisfy our settings, and therefore we link quadratic problems with an exact sparsity term $\Vert {{\mathsf {x}}}\Vert _0$ to copositive optimization. The covered problem class includes sparse least-squares regression under linear constraints, for instance. Numerical comparisons between our method and other approximations are reported from the perspective of the objective function value.

## Introduction

### Motivation

Many real-world applications can be modelled by (nonconvex) quadratic optimization problems with complementarity constraints (QPCCs) [35]. Due to nonconvexity of the objective function and the complementarity constraints, QPCCs form an NP-hard class of problems. Denoting by ${{\mathbb {R}}}^n$ the *n*-dimensional Euclidean space, by ${{\mathbb {R}}}^n_+=\{ {{\mathsf {x}}}\in {{\mathbb {R}}}^n: x_i \ge 0 \text{ for } \text{ all } i\}$ its positive orthant, and by ${{\mathbb {R}}}^{m\times n}$ the set of all real $m\times n$ matrices, we study in this paper the following problem where ${{\mathsf {Q}}}\in {{\mathbb {R}}}^{n\times n}$, ${{\mathsf {c}}}\in {{\mathbb {R}}}^n$, ${{\mathsf {x}}}\in {{\mathbb {R}}}^n$, ${{\mathsf {A}}}\in {{\mathbb {R}}}^{m\times n}$, ${{\mathsf {b}}}\in {{\mathbb {R}}}^m$ and ${{\mathsf {F}}}\in {{\mathbb {R}}}^{n\times n}_+$:

1 $$\begin{aligned} \begin{aligned} \min _{{{\mathsf {x}}}\in {{\mathbb {R}}}^n_+}\quad&{{\mathsf {x}}}^\top {{\mathsf {Q}}}{{\mathsf {x}}}+2{{\mathsf {c}}}^\top {{\mathsf {x}}}\\ \text{ s.t. } \quad&{{\mathsf {A}}}{{\mathsf {x}}}={{\mathsf {b}}}\, , \, {{\mathsf {x}}}^\top {{\mathsf {F}}} {{\mathsf {x}}}= 0\, , \end{aligned} \end{aligned}$$

where the sign $\hbox {}^\top$ denotes transposition. Since all $F_{ij}\ge 0$, observe that $F_{ii} >0$ would entail $x_i^2=0$ for all feasible ${{\mathsf {x}}}$, so that this variable can be dropped. Hence we may and do assume without loss of generality that all $F_{ii}=0$; likewise we may and do assume symmetry: ${{\mathsf {F}}}^\top = {{\mathsf {F}}}$ and ${{\mathsf {Q}}}^\top ={{\mathsf {Q}}}$ as well. Finally, we assume ${{\mathsf {F}}}\ne {{\mathsf {O}}}$.

Problem (1) actually covers a wide range of relevant problems, including, for instance, all mixed-binary quadratic optimization problems (MBQPs) which are hard as well [1, 20]:

$$\begin{aligned} \begin{aligned} \min _{{{\mathsf {x}}}\in {{\mathbb {R}}}^n_+}\quad&{{\mathsf {x}}}^\top {{\mathsf {Q}}}{{\mathsf {x}}}+2{{\mathsf {c}}}^\top {{\mathsf {x}}}\\ \text{ s.t. } \quad&{{\mathsf {A}}}{{\mathsf {x}}}={{\mathsf {b}}}\, , \, x_i \in \{0,1\}\, , \text{ all } i\in I \,, \end{aligned} \end{aligned}$$

with *I* a suitable subset of all coordinate indices.

One of possible approaches to tackle problem (1) is conic relaxation. In particular, [15] proposed a conic relaxation model, which links QPCCs to conic linear optimization problems over the so-called completely positive cone, and which is exact (i.e., a conic reformulation) under some conditions. However, these conditions are not necessarily satisfied in many real-world applications including quadratic optimization problems with a true sparsity term $\Vert {{\mathsf {x}}}\Vert _0$ (see below). The purpose of the paper is to generalize the results of [15] such that the exact relaxation technique is applicable, for instance, to all sparse QPs.

Sparsity of a vector ${{\mathsf {y}}}\in {{\mathbb {R}}}^n$ usually means that the *zero-(pseudo-)norm* which counts the number of non-zero entries $y_i$, i.e.,

$$\begin{aligned} \Vert {{\mathsf {y}}}\Vert _{0}:= \sum _{i=1}^n |\text{ sign } (y_i)|\, , \end{aligned}$$

is small, where

$$\begin{aligned} \text{ sign } (t) := {\left\{ \begin{array}{ll} 1\, , &{}\quad \text{ if } t>0\, ,\\ 0\, , &{}\quad \text{ if } t=0\, ,\\ -1\, , &{}\quad \text{ if } t<0\, .\\ \end{array}\right. } \end{aligned}$$

Moreover, a vector ${{\mathsf {y}}}$ is said to be *k*-*sparse* if $\Vert {{\mathsf {y}}}\Vert _0 = k$. We also recall that for $q>0$, the *q*-*(pseudo-)norms* are defined as

$$\begin{aligned} \Vert {{\mathsf {y}}}\Vert _q := \left[ \sum _{i=1}^n |y_i|^q \right] ^{1/q}\, . \end{aligned}$$

### Literature account (sparse...) and our contribution

Recently, there have been several attempts to solve (1) by a conic approach. For instance [21] employs a doubly nonnegative relaxation and applies a branch-and-bound procedure combined with an augmented Lagrangian approach, assuming convexity of the objective. In a different setting, [39] treats (1) under the assumption that the feasible set is bounded. The same assumption is needed in [30] where the authors solve a conic relaxation with a proximal ADMM variant. In this study however, we present methods for non-convex objectives on unbounded feasible sets.

The literature on sparse optimization and modelling is vast. The majority of applicable formulations replace the discontinuous (thus nonconvex, nonsmooth and non-Lipschitz) term $\Vert \cdot \Vert _0$ by some approximations. To pursue sparsity, these approximations are either employed in constraints or else in an additive regularization term in the objective, involving a trade-off parameter. Such approximate terms include, but are not limited to, *q*-pseudonorms $\Vert \cdot \Vert _{q}$ ($0<q<1$), *p*-norms $\Vert \cdot \Vert _{p}$ ($p\ge 1$), or some combinations of them like $\Vert \cdot \Vert _1-\Vert \cdot \Vert _2$. A subjective sparse selection of related references is [4, 6, 17, 19, 27, 32, 34, 37, 38].

Among the few papers involving the ‘true’ sparsity term $\Vert \cdot \Vert _0$ the likely closest to our proposal is [7]. There the authors use a mixed-binary conic formulation and treat the resulting nonlinear semidefinite optimization problem with a cutting-plane method involving several big-M constants; see also [18] for potential limitations of using similar mixed-integer approaches. Interestingly enough, most of the papers using purely continuous reformulations of problems involving $\Vert \cdot \Vert _0$ in an explicit way, either in the objective or in the constraint, focus on local solutions via NLP solvers, with considerable success, demonstrated by convergence results and impressive empirical findings; see, e.g. [14, 22, 25, 36].

However, questions of global optimality or bounds within this purely continuous model framework seem to have not been addressed before, to the best of our knowledge. The present study tries to narrow this research gap by copositive optimization, yielding rigorous bounds of high quality, albeit at the expense of increased computational cost. It is our hope that in the near future, this cost can be considerably reduced by tapping on recent advances in tractable approximations of copositive optimization problems.

This paper offers:

- a resulting conic relaxation of (1) which is exact under conditions that significantly generalize the classical approach (Burer’s key condition);
- the exactness conditions involve only the constraints and avoid any assumptions on the objective; rather, we show that they yield some implications for the objective which may be hard to test directly;
- some conditions ensuring strong conic duality;
- an approach avoiding discrete variables, use of branching or big constants, by focussing on a purely continuous (in fact, quadratic with linear constraints and one complementarity constraint) exact model of sparse optimization including the ‘true’ sparsity term $\Vert \cdot \Vert _0$; this is important for the efficient performance of implementations, see [5] for a related observation with linear objective functions;
- some experimental evidence on the quality comparison between direct use of $\Vert \cdot \Vert _0$ and the most common approximations.

Let us sketch this in a bit more detail: instead of tackling (1) directly, we adopt copositive relaxation techniques [10, 15] to convexify the targeted problem and end up with a linear conic problem. For surveys on copositive optimization readers are referred to [8, 11, 13, 16, 23, 24, 29]. Note that we propose a refined copositive formulation compared to the general approach via Burer’s result [15]. The latter would result in additional constraints potentially detrimental to algorithmic performance.

As already observed by [3], this relaxation cannot always be tight for problems of the form (1). Here we propose two widely applicable sufficient conditions on (1), ensuring the exactness of the relaxation. To demonstrate the procedure, an application to pursuing sparse solutions for quadratic problems is presented and applied to sparse linear regression, both with and without additional linear (in)equality constraints.

Note the contrast to previous exactness conditions for conic QPCC reformulations: those proposed in [9] for general quadratically constrained quadratic problems (QCQP) need a Slater-type condition on the linear constraints which typically is violated by sparse feasible solutions, so they cannot be applied directly. Those in [3] involve a copositivity condition on the objective which may be hard to test, in particular since the involved cone may be non-convex. On the other hand, [3] shows how to reformulate a general cone-constrained QCQP into another cone-constrained QPCC. As it turns out, even if the original cone constraints are linear, the reformulated cone constraints suggested by [3] involve second-order cones, which make copositivity tests even harder. It should be stressed that one of the merits of [3] lies in clarification of the underlying general structure. However, for practical purposes it seems justified to treat the particular case of QPCC among the generic QCQPs separately, exploiting the special circumstances.

Contrasting most of the studies in literature, we directly use the discontinuous (thus nonconvex, nonsmooth and non-Lipschitz) term $\Vert \cdot \Vert _0$ to pursue sparsity, rather than using common approximations. While several conditions are proposed in the literature under which some approximations of $\Vert \cdot \Vert _0$ yield indeed sparsity, up to now it remained largely unclear how good all above mentioned approximations are in general, compared to $\Vert \cdot \Vert _0$, in terms of ability of gaining sparsity without losing accuracy of the original problem. Here we will justify our approach by help of a small experimental study, where we compare the optimal values between our method and several recently popular approximate methods on a test set of randomly generated problems.

### Paper organization and notation

The paper is arranged as follows. In Sect. 2, as a significant extension to Burer’s results, we propose two sufficient conditions ensuring the exactness of copositive relaxation for QPCCs. As shown in [25] and detailed in Sect. 3.1, the problem of finding sparse solutions for quadratic optimization problems turns out to be a QPCC satisfying our proposed conditions, and therefore an exact copositive relaxation is obtained. The same holds true for convex objectives under hard sparsity constraints, as observed already in [14]; see Sect. 3.2. Both regimes (significantly different as noted, e.g., by [36]) are met in the case of sparse linear regression treated in Sect. 3.3. In Sect. 4, we compare our method with several approximate methods nowadays popular for sparse quadratic problems, from the perspective of optimal value.

Throughout the paper, by bold lower case, e.g. ${{\mathsf {x}}}$, and bold upper case letter, e.g. ${{\mathsf {X}}}$, we denote a vector and a matrix respectively. For instance, ${{\mathsf {o}}}$ and ${{\mathsf {O}}}$ stand for zero vectors and matrices of suitable size, respectively. For any integers *a*, *b* we denote by $[a\! : \! b]$ the set of all integers *k* satisfying $a\le k \le b$. For ${{\mathsf {x}}}\in {{\mathbb {R}}}^n$, the *n*-dimensional Euclidean space, and any nonempty $I\subset [1\!:\!n]$, we denote by ${{\mathsf {x}}}_I = [x_i]_{i\in I}$ the restricted subvector.

By $\langle {{{\mathsf {X}}}} , {{{\mathsf {Y}}}} \rangle := \sum _{i=1}^{n} \sum _{1}^{n} x_{ij} y_{ij}$ we denote the inner product between symmetric matrices. We also define sets by:

$$\begin{aligned} \begin{aligned} \Delta ^n&= \{{{\mathsf {x}}}\in {\mathbb {R}}_{+}^n: \Vert {{\mathsf {x}}}\Vert _1 = 1\}\, , \\ {\mathcal {S}}^n&= \{{{\mathsf {X}}}\in {\mathbb {R}}^{n\times n}: {{\mathsf {X}}}= {{\mathsf {X}}}^\top \}\, ,\\ {\mathcal {N}}^n&= {\mathcal {S}}^n \cap {\mathbb {R}}_{+}^{n\times n}\, ,\\ {\mathcal {P}}^n&=\{{{\mathsf {X}}}\in {\mathcal {S}}^n: {{\mathsf {v}}}^\top {{\mathsf {X}}}{{\mathsf {v}}}\ge 0 \text{ for } \text{ all } {{\mathsf {v}}}\in {\mathbb {R}}^{n}\},\\ \mathcal {COP}^n&= \{{{\mathsf {X}}}\in {\mathcal {S}}^n: {{\mathsf {v}}}^\top {{\mathsf {X}}}{{\mathsf {v}}}\ge 0 \text{ for } \text{ all } {{\mathsf {v}}}\in {\mathbb {R}}_{+}^{n}\}\, ,\\ {\mathcal{CP}\mathcal{}}^n&=\{\sum _{i\in I}{{\mathsf {x}}}_i{{\mathsf {x}}}_i^\top \in {\mathcal {S}}^n: {{\mathsf {x}}}_i \in {\mathbb {R}}_{+}^{n}\, , \text{ for } \text{ all } i \in I\}\, . \end{aligned} \end{aligned}$$

For a given matrix ${{\mathsf {Q}}}\in {\mathcal {S}}^n$, ${{\mathsf {q}}}_i^\top$ presents the *i*-th row of ${{\mathsf {Q}}}$ and $q_{ij}$ stands for the *ij*-th entry. Particularly, by ${{\mathsf {e}}}_i\in {{\mathbb {R}}}^n$ we mean the *i*-th column of the $n\times n$ identity matrix, and by ${{\mathsf {e}}}=\sum _{i=1}^n {{\mathsf {e}}}_i$ the all-ones vector in ${{\mathbb {R}}}^n$, so that $\Delta ^n = \{ {{\mathsf {x}}}\in {{\mathbb {R}}}^n_+ : {{\mathsf {e}}}^\top {{\mathsf {x}}}=1\}$.

## Conic reformulation of QPCCs

### Conic formulation: Burer’s key condition

As announced, we focus on QPCCs of the form

P $$\begin{aligned} \begin{aligned} z_P^*:= \inf _{{{\mathsf {x}}}\in {\mathbb {R}}^n_+} \quad&f({{\mathsf {x}}}):= {{\mathsf {x}}}^\top {{\mathsf {Q}}} {{\mathsf {x}}}+2{{\mathsf {c}}}^\top {{\mathsf {x}}}\\ s.t. \quad&{{\mathsf {a}}}_i ^\top {{\mathsf {x}}}=b_i\, ,\quad i{ \in \! [{1}\! : \! {m}]}\, ,\\&\ {{\mathsf {x}}}^\top {{\mathsf {F}}} {{\mathsf {x}}}=0\, ,\\ \end{aligned} \end{aligned}$$

where ${{\mathsf {Q}}}\in {{\mathcal {S}}}^n$, ${{\mathsf {F}}}\in {{\mathcal {N}}}^n\setminus \left\{ {{\mathsf {O}}}\right\}$ with zero diagonal, $\left\{ {{\mathsf {c}}},{{\mathsf {a}}}_i\right\} \subset {{\mathbb {R}}}^n$ and ${{\mathsf {b}}}=[b_1,\ldots , b_m]^\top \in {{\mathbb {R}}}^m$. We collect ${{\mathsf {A}}}^\top = [{{\mathsf {a}}}_1,\ldots , {{\mathsf {a}}}_m]$ to get an $m\times n$ matrix ${{\mathsf {A}}}$ and represent the linear constraints in the form

$$\begin{aligned} {{\mathsf {x}}}\in L := \{{{\mathsf {x}}}\in {{\mathbb {R}}}^n_+ : {{\mathsf {A}}}{{\mathsf {x}}}= {{\mathsf {b}}}\}\, . \end{aligned}$$

Problem (P) is an all-quadratic problem of special structure; remark that the case where $\left\{ {{\mathsf {Q}}},{{\mathsf {F}}}\right\} \subset {{\mathcal {P}}}^n$ reduces to a traditional convex QP of the form

$$\begin{aligned} \inf _{{{\mathsf {x}}}\in {\mathbb {R}}^n_+} \left\{ f({{{\mathsf {x}}}}) : {{\mathsf {A}}}{{\mathsf {x}}}= {{\mathsf {b}}}, \, {{\mathsf {F}}}{{\mathsf {x}}}={{\mathsf {o}}}\right\} \, , \end{aligned}$$

which can be solved in polynomial time to arbitrary accuracy. In all other cases, this problem class includes NP-hard nonconvex subclasses. Problems of form (P) have been addressed in the seminal paper by Burer [15] who related (P) to the following conic optimization problem:

C $$\begin{aligned} \begin{aligned} z^*_C:= \min \quad&g({{\mathsf {Y}}}):=\langle {{{\mathsf {Q}}}} , {{{\mathsf {X}}}} \rangle + 2{{\mathsf {c}}}^\top {{\mathsf {x}}}\\ s.t. \quad&{{\mathsf {a}}}_{i}^{\top }{{\mathsf {x}}}= b_{i}, \quad i{ \in \! [{1}\! : \! {m}]}\, ,\\&{{\mathsf {a}}}_{i}^\top {{\mathsf {X}}} {{\mathsf {a}}}_{i} = b^{2}_{i}\, , \quad i{ \in \! [{1}\! : \! {m}]}\, ,\\&\langle {{{\mathsf {F}}}} , {{{\mathsf {X}}}} \rangle = 0\, ,\\&{{\mathsf {Y}}}:= \begin{pmatrix} 1 &{}{{\mathsf {x}}}^\top \\ {{\mathsf {x}}}&{}{{\mathsf {X}}}\\ \end{pmatrix} \in {\mathcal{CP}\mathcal{}}^{n+1}\, .\\ \end{aligned} \end{aligned}$$

The following *horizon cone* of *L* plays a crucial role in this analysis:

$$\begin{aligned} L_\infty := \left\{ {{\mathsf {x}}}\in {{\mathbb {R}}}^n_+ : {{\mathsf {A}}}{{\mathsf {x}}}={{\mathsf {o}}}\right\} \end{aligned}$$

The set of indices belonging to variables which remain bounded over *L*,

2 $$\begin{aligned} B:=\left\{ i{ \in \! [{1}\! : \! {n}]} : \text{ there } \text{ is } \text{ an } M_i >0 \text{ such } \text{ that } x_i \le M_i \text{ if } {{\mathsf {x}}}\in L\right\} \, . \end{aligned}$$

is related to $L_\infty$ by the implication ${{\mathsf {z}}}\in L_\infty \Longrightarrow {{\mathsf {z}}}_B = {{\mathsf {o}}}$. We need one more index set, namely

$$\begin{aligned} S_{{\mathsf {F}}}:= \left\{ i{ \in \! [{1}\! : \! {n}]} : F_{ij} > 0 \text{ for } \text{ some } j{ \in \! [{1}\! : \! {n}]}\right\} \, , \end{aligned}$$

the projection of the *support* of ${{\mathsf {F}}}$,

$$\begin{aligned} {\mathcal {E}}:= \left\{ \left\{ i,j\right\} : F_{ij} > 0 \right\} \, . \end{aligned}$$

We will view the sparsity pattern of ${{\mathsf {F}}}$ as an undirected graph ${{\mathcal {G}}}_{{\mathsf {F}}}:= (V,{\mathcal {E}})$ on the vertex set $V=[1\!:\!n]$ and edge set ${\mathcal {E}}$. Since by assumption ${{\mathsf {F}}}\in {{\mathcal {N}}}^n$, we have ${{\mathsf {x}}}^\top {{\mathsf {F}}} {{\mathsf {x}}}= 0$ if and only if $x_i x_j = 0$ for all $\left\{ i,j\right\} \in {\mathcal {E}}$, so ${{\mathcal {G}}}_{{\mathsf {F}}}$ comprises all information on ${{\mathsf {F}}}$ relevant to (P). Thus we can always replace ${{\mathsf {F}}}$ by ${{\mathsf {E}}}:=\sum \limits _{\left\{ i,j\right\} \in {\mathcal {E}}} {{\mathsf {e}}}_i{{\mathsf {e}}}_j^\top$ without changing the problem.

With these notational preliminaries, we easily can formulate the so-called *key condition* put forward by Burer [15] in the analysis of QPCCs:

K $$\begin{aligned} S_{{\mathsf {F}}}\subseteq B\, , \end{aligned}$$

in other words, any variable $x_j$ actually involved in the complementarity constraints, must remain bounded over the linear portion *L* of the feasible set. Under (K), Burer proved that the conic relaxation (C) of (P) is exact.

### Relaxing the key condition: vertex covers

Unfortunately, condition (K) is not necessarily met in some relevant applications, and in other applications with a convex objective, the key condition is superfluous (even if the problem is not convex as may happen for ${{\mathsf {F}}}\notin {{\mathcal {P}}}^n$). In this section, we will propose less restrictive conditions ensuring exactness which can be successfully applied to several optimization problems, including sparse least-squares regression with soft or hard sparsity constraints, a hot topic in data science.

Given a graph ${{\mathcal {G}}}= (V,{\mathcal {E}})$, recall that a subset $C\subseteq V$ is said to be a *vertex cover* for ${{\mathcal {G}}}$ if $\left\{ i,j\right\} \cap C\ne \emptyset$ for all $\left\{ i,j\right\} \in {\mathcal {E}}$ (this holds in particular if $\left\{ C,V\setminus C\right\}$ is a bipartition of ${{\mathcal {G}}}$ in the sense that $(i,j)\in C\times (V\setminus C) \cup (V\setminus C)\times C$ for any $\{i,j\}\in {\mathcal {E}}$, but more general vertex covers *C* may also have insider edges, $C\times C\cap {\mathcal {E}}\ne \emptyset$). Obviously, *V* itself is a vertex cover but it may be too large for our purposes, as we aim to replace Burer’s $S_{{\mathsf {F}}}$ with a (typically smaller) vertex cover *C* for ${{\mathcal {G}}}_{{\mathsf {F}}}$.

The *relaxed key condition* reads as follows, using notation (2):

R $$\begin{aligned} \text{ There } \text{ is } \text{ a } \text{ vertex } \text{ cover } C\subseteq B \text{ for } {{\mathcal {G}}}_{{\mathsf {F}}} \text{ and } \text{ a } \text{ point } {{\hat{{{\mathsf {x}}}}}}\in L \text{ with } {{\hat{{{\mathsf {x}}}}}}_C={{\mathsf {o}}}\, , \end{aligned}$$

or equivalently, there is a vertex cover *C* for ${{\mathcal {G}}}_{{\mathsf {F}}}$ and some (P)-feasible ${{\hat{{{\mathsf {x}}}}}}$ such that for all $j\in C$ there exists an $M_j>0$ such that

$$\begin{aligned} {{\hat{x}}}_j = 0\quad \text{ and } \quad x_j \le M_j \text{ for } \text{ all } {{\mathsf {x}}}\in L \,. \end{aligned}$$

Obviously, the conditions in (R) are met more easily if *C* is smaller, so the system ${\mathfrak {C}}({{\mathsf {F}}})$ in which we collect all *minimal vertex covers*^1^ for ${{\mathcal {G}}}_{{\mathsf {F}}}$ is of particular relevance. But to keep our approach more transparent, we will focus on one vertex cover *C* first (which is sufficient for application to sparse regression) and later on, extend the following conditions for future applications to more general QPCCs. First let us note that indeed (R) relaxes (K):

#### Proposition 1

Assume that (P) is feasible, so that there exists a point ${{\hat{{{\mathsf {x}}}}}}\in L$ such that ${{\hat{{{\mathsf {x}}}}}}^\top {{\mathsf {F}}} {{\hat{{{\mathsf {x}}}}}}= 0$. Then (K) implies (R).

#### Proof

We show that $C:=\left\{ j\in S_{{\mathsf {F}}}: {\hat{x}}_j = 0\right\}$ and ${{\hat{{{\mathsf {x}}}}}}\in L$ satisfy (R) under condition (K). Indeed, by definition ${{\hat{{{\mathsf {x}}}}}}_C= {{\mathsf {o}}}$ and $C\subseteq S_{{\mathsf {F}}}$. Furthermore, ${{\hat{{{\mathsf {x}}}}}}^\top {{\mathsf {F}}} {{\hat{{{\mathsf {x}}}}}}= 0$ implies ${\hat{x}}_i{\hat{x}}_j = 0$ for all $\left\{ i,j\right\} \in {\mathcal {E}}$, so that $\left\{ i,j\right\} \cap C \ne \emptyset$, hence *C* is a vertex cover for ${{\mathcal {G}}}_{{\mathsf {F}}}$. $\square$

In case of a convex objective, condition (K) and even the condition (R) are unnecessary. Actually, we only need the conclusion that ${{\mathsf {Q}}}$ is copositive on the cone

$$\begin{aligned} L_\infty \cap F :=\left\{ {{\mathsf {z}}}\in {{\mathbb {R}}}^n_+ : {{\mathsf {A}}}{{\mathsf {z}}}={{\mathsf {o}}} \text{ and } {{\mathsf {z}}}^\top {{\mathsf {F}}} {{\mathsf {z}}}= 0\right\} \, , \end{aligned}$$

the intersection of the horizon cone of *L* with the complementarity set

$$\begin{aligned} F:=\left\{ {{\mathsf {x}}}\in {{\mathbb {R}}}^n_+ : {{\mathsf {x}}}^\top {{\mathsf {F}}} {{\mathsf {x}}}= 0\right\} \, . \end{aligned}$$

Condition (R) guarantees that $L_\infty \cap F = L_\infty$ and the mentioned copositivity property of ${{\mathsf {Q}}}$, which is also implied by convexity of the objective. Essential arguments are summarized in the following.

#### Lemma 2

Assume $z_P^*>-\infty$, so that problem (P) is bounded, and that (R) holds. Then $L_\infty \subseteq F$, and for any ${{\mathsf {z}}}\in L_\infty$ we have ${{\mathsf {z}}}^\top {{\mathsf {Q}}} {{\mathsf {z}}}\ge 0$. The latter property holds if ${{\mathsf {Q}}}\in \mathcal {COP}^n$ (e.g., because ${{\mathsf {Q}}}$ is positive-semidefinite).

#### Proof

If condition (R) holds for some vertex cover $C\subseteq V$ of ${{\mathcal {G}}}_{{\mathsf {F}}}$, pick an arbitrary ${{\mathsf {z}}}\in L_\infty$ and select ${{\hat{{{\mathsf {x}}}}}}\in L$ with ${{\hat{{{\mathsf {x}}}}}}_C = {{\mathsf {o}}}$. Then for all $t\ge 0$, we have ${{\mathsf {x}}}(t)={{\hat{{{\mathsf {x}}}}}}+t\,{{\mathsf {z}}}\in L$ which implies, by $C\subseteq B$ from (R), that ${{\mathsf {z}}}_C= {{\mathsf {o}}}$, which in turn implies ${{\mathsf {x}}}(t)_C={{\mathsf {o}}}$ for all $t\ge 0$. So $x_i(t)x_j(t)=0$ for all $j\in C$ (and all $i\in V$) which yields ${{\mathsf {x}}}(t)^\top {{\mathsf {F}}} {{\mathsf {x}}}(t)=0$ since *C* is a vertex cover for ${{\mathcal {G}}}_{{\mathsf {F}}}$. Hence ${{\mathsf {x}}}(t)\in F$ and in a similar fashion, we derive ${{\mathsf {z}}}\in F$. We conclude $L_\infty \subseteq F$ and furthermore that ${{\mathsf {x}}}(t)$ is (P)-feasible, hence

$$\begin{aligned} -\infty < z_P^* \le f({{\mathsf {x}}}(t)) = f({{\hat{{{\mathsf {x}}}}}}) + 2t\, {{\mathsf {z}}}^\top ({{\mathsf {Q}}}{{\hat{{{\mathsf {x}}}}}}+ {{\mathsf {c}}}) + t^2\, {{\mathsf {z}}}^\top {{\mathsf {Q}}} {{\mathsf {z}}}\quad \text{ for } \text{ all } t\ge 0\, , \end{aligned}$$

which implies ${{\mathsf {z}}}^\top {{\mathsf {Q}}} {{\mathsf {z}}}\ge 0$. This implication is trivial if ${{\mathsf {Q}}}\in \mathcal {COP}^n$, since $L_\infty \subseteq {{\mathbb {R}}}^n_+$ by definition. $\square$

Note: if ${{\mathsf {z}}}^\top {{\mathsf {Q}}} {{\mathsf {z}}}= 0$, we could infer, by the same reasoning, that ${{\mathsf {z}}}^\top \nabla f({{\hat{{{\mathsf {x}}}}}}) = 2 {{\mathsf {z}}}^\top ({{\mathsf {Q}}}{{\hat{{{\mathsf {x}}}}}} + {{\mathsf {c}}})\ge 0$, i.e., that ${{\mathsf {z}}}$ could not be a strict descent direction for *f* at ${{\hat{{{\mathsf {x}}}}}}$. But we will not explore this first-order information further here.

Now we formulate a variant applicable to other QPCCs:

#### Lemma 3

Assume $z_P^*>-\infty$, so that problem (P) is bounded. Suppose that for any $C \in {\mathfrak {C}}({{\mathsf {F}}})$ with nontrivial cone $\left\{ {{\mathsf {x}}}\in L_\infty : {{\mathsf {x}}}_{C} = {{\mathsf {o}}}\right\}$, there exists a point ${{\hat{{{\mathsf {x}}}}}}\in L$ such that ${{\hat{{{\mathsf {x}}}}}}_C={{\mathsf {o}}}$. Then ${{\mathsf {Q}}}$ is $(L_\infty \cap F)$-copositive.

#### Proof

First observe that

$$\begin{aligned} F \subseteq \bigcup _{C\in {\mathfrak {C}}({{\mathsf {F}}})} \{{{\mathsf {z}}}\in {{\mathbb {R}}}^n_+ : {{\mathsf {z}}}_C = {{\mathsf {o}}}\}\, . \end{aligned}$$

Indeed, for any ${{\mathsf {z}}}\in F$ consider $C_{{\mathsf {z}}}=\left\{ i\in V: z_i=0\right\} \ne \emptyset$ (due to ${{\mathsf {F}}}\ne {{\mathsf {O}}}$), which obviously is a vertex cover for ${{\mathcal {G}}}_{{\mathsf {F}}}$. Clearly there is an $C\in {\mathfrak {C}}({{\mathsf {F}}})$ with $C\subseteq C_{{\mathsf {z}}}$, so ${{\mathsf {z}}}_C = {{\mathsf {o}}}$. Then the result follows by arguments similar to those employed in the previous proof. $\square$

The following examples shall demonstrate wider applicability of our proposed conditions. But for the readers’ convenience let us start with a counterexample already presented in [3] which shows that some conditions are needed to avoid a positive, or even infinite, conic relaxation gap:

#### Example 1

3 $$\begin{aligned} \begin{aligned} \min -x_2^2\\ \text{ s.t. }\quad x_1 + x_2 - x_3&= 3\, ,\\ x_1 - x_4&=2\, ,\\ x_1 x_2&= 0\, ,\\ {{\mathsf {x}}}&\in {{\mathbb {R}}}^4_+\, . \end{aligned} \end{aligned}$$

As argued in [3, Ex.1], the relaxation (C) is unbounded below while the optimal value of (P) is zero. As can be seen easily, conditions (K) and (R) are violated, and as well the one of Lemma 3: all ${{\mathsf {x}}}\in L$ must have $x_1\ge 2>0$, so ${{\mathsf {x}}}_C\ne {{\mathsf {o}}}$ for $C=\left\{ 1\right\} \in {\mathfrak {C}}({{\mathsf {F}}})$ while $L_\infty$ contains, e.g., $(0,1,1,0)^\top$.

#### Example 2

4 $$\begin{aligned} \begin{aligned} x_1 - x_2 - x_3 + x_4&= 1\, ,\\ x_1 + x_2&=1\, ,\\ x_1 x_2 + x_1 x_3&= 0\, ,\\ {{\mathsf {x}}}&\in {{\mathbb {R}}}^4_+\, . \end{aligned} \end{aligned}$$

Note that the second constraint implies boundedness of $x_1$ and $x_2$, so we have $B = \{1,2\}$. However, since the set *S* of variables involved in the complementary constraint is $S= \{1,2,3\}\supset \{1,2\}$, condition (K) is violated. On the other hand, the class of minimal vertex covers of (4) is ${\mathfrak {C}}({{\mathsf {F}}})=\{\{1\},\{2,3\}\}$, with a vertex cover $C = \{1\}\subset B$. Moreover, $(0,1,1,3)^\top$ is (4)-feasible and therefore condition (R) is met. Also, the condition of Lemma 3 holds, as $C=\left\{ 2,3\right\}$ yields a trivial cone $\left\{ {{\mathsf {z}}}\in L_\infty : {{\mathsf {z}}}_{C} = {{\mathsf {o}}}\right\}$.

#### Example 3

5 $$\begin{aligned} \begin{aligned} x_1 - x_2 - x_3 + x_4 -x_5&= 1\, ,\\ x_1 + x_2&=1\, ,\\ x_2 x_3 + x_3 x_4&= 0\, ,\\ {{\mathsf {x}}}&\in {{\mathbb {R}}}^5_+\, . \end{aligned} \end{aligned}$$

As in (4), we have $B = \{1,2\}$. Again, condition (K) is violated since $S=\{2,3,4\}$ is not contained in *B*. The class of minimal vertex covers of (5) is ${\mathfrak {C}}({{\mathsf {F}}})=\{\{3\},\{2,4\}\}$. None of them is contained in *B*, so condition (R) is violated. For $C=\{3\}$, the cone

$$\begin{aligned} \{{{\mathsf {x}}}\in L_{\infty } : {{\mathsf {x}}}_{C} = {{\mathsf {o}}}\} = \{{{\mathsf {x}}}\in {{\mathbb {R}}}^5_+: x_4 =x_5, x_1=x_2=x_3 = 0\} \end{aligned}$$

is nontrivial, and the point ${{\hat{{{\mathsf {x}}}}}} = (1,0,0,0,0)^\top \in L$ satisfies ${{\hat{{{\mathsf {x}}}}}}_C ={{\mathsf {o}}}$. But for $C = \{2,4\}$, the cone

$$\begin{aligned} \{{{\mathsf {x}}}\in L_{\infty } : {{\mathsf {x}}}_{C} = {{\mathsf {o}}}\}=\{{{\mathsf {x}}}\in {{\mathbb {R}}}^5_+ : x_3 +x_5 =0, x_1=x_2=x_4 = 0 \}= \left\{ {{\mathsf {o}}}\right\} \, . \end{aligned}$$

Therefore the system satisfies the condition in Lemma 3.

#### Example 4

6 $$\begin{aligned} \begin{aligned} x_1 + x_4 - x_5 + x_6&= 5\, ,\\ x_2 + x_3 - x_5 + x_6&= 1\, ,\\ x_1 + x_2&=1\, ,\\ x_1 x_3 + x_1 x_4&= 0\, ,\\ {{\mathsf {x}}}&\in {{\mathbb {R}}}^6_+\, . \end{aligned} \end{aligned}$$

As in (4), we have $B = \{1,2\}$. Again, condition (K) is violated since $S=\{1,3,4\}$ is not contained in *B*. On the other hand, the class of minimal vertex covers of (6) is ${\mathfrak {C}}({{\mathsf {F}}})=\{\{1\},\{3,4\}\}$, with a vertex cover $C = \{1\}\subset B$. Moreover, $(0,1,0,5,2,2)^\top$ is (6)-feasible and therefore condition (R) is met. For $C=\{3,4\}$, the cone

$$\begin{aligned} \{{{\mathsf {x}}}\in L_{\infty } : {{\mathsf {x}}}_{C} = {{\mathsf {o}}}\} = \{{{\mathsf {x}}}\in {{\mathbb {R}}}^6_+: x_5 =x_6, x_1=x_2=x_3=x_4 = 0\} \end{aligned}$$

is nontrivial. However, the set $\{{{\mathsf {x}}}\in L : {{\mathsf {x}}}_{C} = {{\mathsf {o}}}\}$ is empty now, because ${{\mathsf {x}}}_C = {{\mathsf {o}}}$ implies a contradiction, looking at the sum of the first two constraints and the third one, in conjunction with the sign constraints on $x_1$ and $x_2$. Therefore, the condition of Lemma 3 is violated.

While (K) implies (R), it does not imply the condition of Lemma 3; we give the following example:

#### Example 5

7 $$\begin{aligned} \begin{aligned} x_3 - x_4 + x_5&= 2\, ,\\ x_2 - x_4 + x_5&= 1\, ,\\ x_1 + x_2 + x_3&=2\, ,\\ x_1 x_2 + x_1 x_3&= 0\, ,\\ {{\mathsf {x}}}&\in {{\mathbb {R}}}^5_+\, . \end{aligned} \end{aligned}$$

Since we have $S=\{1,2,3\} = B$, condition (K) holds. On the other hand, the class of minimal vertex covers of (7) is ${\mathfrak {C}}({{\mathsf {F}}})=\{\{1\},\{2,3\}\}$. For $C=\{2,3\}$, the cone

$$\begin{aligned} \{{{\mathsf {x}}}\in L_{\infty } : {{\mathsf {x}}}_{C} = {{\mathsf {o}}}\} = \{{{\mathsf {x}}}\in {{\mathbb {R}}}^5_+: x_4 =x_5, x_1=x_2=x_3= 0\} \end{aligned}$$

is nontrivial. However, the set $\{{{\mathsf {x}}}\in L : {{\mathsf {x}}}_{C} = {{\mathsf {o}}}\}$ is empty now because of the contradiction between the first two constraints. Therefore, the condition of Lemma 3 is violated.

As a final note, it is easy to construct examples for which both (K) and the condition of Lemma 3 are satisfied [3].

Now we are in a position to state and prove the following result which generalizes the one in [15]. Although the main arguments are well known by now, we repeat them here for the readers’ convenience. Note that even without any assumptions, the first part of the following proof also shows that (P) is feasible if (C) is feasible.

#### Theorem 4

Suppose that ${{\mathsf {Q}}}$ is $(L_\infty \cap F)$-copositive in the sense of Lemmas 2, 3. Then the QPCC problem (P) is equivalent to the conic problem (C).

#### Proof

It is easy to see that any (P)-feasible ${{\mathsf {x}}}\in {{\mathbb {R}}}^n_+$ gives rise to a (C)-feasible point ${{\mathsf {Y}}}= {{\mathsf {u}}}{{\mathsf {u}}}^\top \in {{\mathcal {C}}}{{\mathcal {P}}}^{n+1}$ of rank one with ${{\mathsf {u}}}^\top =[1,{{\mathsf {x}}}^\top ]$ and same objective value $f({{\mathsf {x}}})=\langle {{{\mathsf {Q}}}} , {{{\mathsf {x}}}{{\mathsf {x}}}^\top } \rangle + 2{{\mathsf {c}}}^\top {{\mathsf {x}}}= g({{\mathsf {Y}}})$. Therefore (C) is a conic relaxation of (P) and $z_C^* \le z_P^*$. We proceed to show equivalence by establishing the reverse inequality. To this end, take a (C)-feasible and optimal ${{\mathsf {Y}}}$ (or approximately optimal, in case of non-attainability for either of the problems (P) or (C); we will address primal attainability later). Then consider the following decomposition for this completely positive matrix:

8 $$\begin{aligned} {{\mathsf {Y}}}= \begin{pmatrix}1&{}{{\mathsf {x}}}^{\top }\\ {{\mathsf {x}}}&{}{{\mathsf {X}}}\end{pmatrix}=\sum _{k\in K} \begin{pmatrix}\lambda _{k}\\ {{\mathsf {z}}}_k\end{pmatrix} \begin{pmatrix}\lambda _{k}\\ {{\mathsf {z}}}_k\end{pmatrix}^{\top },\quad \lambda _{k}\in {\mathbb {R}}_{+},\ {{\mathsf {z}}}_k\in {\mathbb {R}}_{+}^{n},\, k\in K\, , \end{aligned}$$

with $g({{\mathsf {Y}}}) = \langle {{{\mathsf {Q}}}} , {{{\mathsf {X}}}} \rangle + 2{{\mathsf {c}}}^\top {{\mathsf {x}}}\approx z_C^*$. Denote by $K_{0}:=\{k\in K:\lambda _{k}=0\}$ and $K_{+}:=\{k\in K:\lambda _{k}>0\}$ respectively. The following equalities hold directly by the decomposition (8) and the constraints in (C):

9 $$\begin{aligned} 1 = \sum _{k\in K}\lambda _{k}^2\, , \quad ( \text{ so } K_+\ne \emptyset )\, , \end{aligned}$$

10 $$\begin{aligned} b_i=a_i^\top {{\mathsf {x}}}= \sum _{k\in K}\lambda _{k}{{\mathsf {a}}}_{i}^{\top }{{\mathsf {z}}}_k,\quad i{ \in \! [{1}\! : \! {m}]}\, , \end{aligned}$$

11 $$\begin{aligned} b_i^2={{\mathsf {a}}}_i^\top {{\mathsf {X}}} {{\mathsf {a}}}_i= \sum _{k\in K}({{\mathsf {a}}}_{i}^{\top }{{\mathsf {z}}}_k)^2, \quad i{ \in \! [{1}\! : \! {m}]}\, . \end{aligned}$$

Combining the equalities (9), (10) and (11), we have for any fixed $i{ \in \! [{1}\! : \! {m}]}$,

$$\begin{aligned} \sum _{k\in K}\lambda _{k}^2\cdot \sum _{k\in K}({{\mathsf {a}}}_{i}^{\top }{{\mathsf {z}}}_k)^2 = 1\cdot b_i^2 = b_i^2 = \left( \sum _{k\in K}\lambda _{k}{{\mathsf {a}}}_{i}^{\top }{{\mathsf {z}}}_k \right) ^2\, . \end{aligned}$$

By the condition of equality case of the Cauchy-Schwarz inequality, there exists $\beta _{i}\in {\mathbb {R}}$ such that

12 $$\begin{aligned} {{\mathsf {a}}}_{i}^{\top }{{\mathsf {z}}}_k=\beta _{i}\lambda _{k} \quad \text{ for } \text{ all } k\in K\, . \end{aligned}$$

Multiplying by $\lambda _k$ and summing relations (12) across *k* and by (9) and (10), we obtain

13 $$\begin{aligned} \beta _{i}=b_{i}\quad \text{ for } \text{ all } i{ \in \! [{1}\! : \! {m}]}\, . \end{aligned}$$

Moreover, by (C)-feasibility of ${{\mathsf {Y}}}$ we have

$$\begin{aligned} 0 = \langle {{{\mathsf {F}}}} , {{{\mathsf {X}}}} \rangle = \sum _{k\in K} {{\mathsf {z}}}_k^\top {{\mathsf {F}}} {{\mathsf {z}}}_k\Longrightarrow {{\mathsf {z}}}_k^\top {{\mathsf {F}}} {{\mathsf {z}}}_k=0 \, , \text{ for } \text{ all } k\in K\, . \end{aligned}$$

Therefore, by taking ${{\mathsf {x}}}_k:=\frac{1}{\lambda _{k}}\, {{{\mathsf {z}}}_k}$ if $\lambda _{k}>0$, we get ${{\mathsf {x}}}_k\in L \cap F$ for all $k\in K_+$ while for $\lambda _{k}=0$ we obtain ${{\mathsf {A}}}{{\mathsf {z}}}_k = {{\mathsf {o}}}$, i.e., ${{\mathsf {z}}}_k\in L_\infty \cap F$ and therefore ${{\mathsf {z}}}_k^\top {{\mathsf {Q}}} {{\mathsf {z}}}_k\ge 0$ for all $k\in K_0$. We obtain that all ${{\mathsf {x}}}_k$ are (P)-feasible, so that

$$\begin{aligned}z_P^* \le z_+^* :=\min \left\{ f({{\mathsf {x}}}_k) : k\in K_+\right\} \le z_C^*\, , \end{aligned}$$

where the last inequality follows from

$$\begin{aligned} \begin{array}{rcl} z_+^* &{}= &{}\sum \limits _{k\in K_+} (\lambda _k)^2 z_+^* \le \sum \limits _{k\in K_+} (\lambda _k)^2 f({{\mathsf {x}}}_k) \\ &{}= &{}\sum \limits _{k\in K_+} \left[ {{\mathsf {z}}}_k^\top {{\mathsf {Q}}} {{\mathsf {z}}}_k+ \lambda _k {{\mathsf {c}}}^\top {{\mathsf {z}}}_k \right] \le \sum \limits _{k\in K_+} \left[ {{\mathsf {z}}}_k^\top {{\mathsf {Q}}} {{\mathsf {z}}}_k+ \lambda _k {{\mathsf {c}}}^\top {{\mathsf {z}}}_k \right] + \sum \limits _{k\in K_0} {{\mathsf {z}}}_k^\top {{\mathsf {Q}}} {{\mathsf {z}}}_k\\ &{}= &{}\sum \limits _{k\in K} \left[ {{\mathsf {z}}}_k^\top {{\mathsf {Q}}} {{\mathsf {z}}}_k+ \lambda _k {{\mathsf {c}}}^\top {{\mathsf {z}}}_k \right] = g({{\mathsf {Y}}}) \approx z_C^*\, , \end{array} \end{aligned}$$

which establishes the desired inequality $z_P^* \le z_C^*$ and moreover that any ${{\mathsf {x}}}^k$ realizing the minimum in $z_+^*$ provides an (approximate) optimal solution to (P). $\square$

Note: since the feasible set of (P) is a finite union of polyhedra (if not empty), the Frank-Wolfe theorem guarantees attainability of $z_P^*$ if this value is finite. Slater’s condition (strict feasibility of the conic dual below) would ensure attainability of $z_C^*$, but above equivalence ensures this automatically: indeed, ${{\mathsf {Y}}}^*= {{\mathsf {u}}}^*({{\mathsf {u}}}^*)^\top$ solves (C) if $({{\mathsf {u}}}^*)^\top = [1,({{\mathsf {x}}}^*)^\top ]$ and ${{\mathsf {x}}}^*$ solves (P).

### A simplification of (C) and its dual

We show a possible simplification of (C) which is initially proposed in [15], reducing the cone in the feasible set from ${\mathcal{CP}\mathcal{}}^{n+1}$ to ${\mathcal{CP}\mathcal{}}^{n}$, and which works under

#### Condition 1

There exists ${{\mathsf {y}}}\in {\mathbb {R}}_{+}^{m}$ such that

$$\begin{aligned} {{\mathsf {d}}}:= {{\mathsf {A}}}^\top {{\mathsf {y}}}= \sum _{i=1}^{m}y_i{{\mathsf {a}}}_i\in {{\mathbb {R}}}^n_+ \quad \text{ and } \quad {{\mathsf {b}}}^\top {{\mathsf {y}}}= 1\, . \end{aligned}$$

Condition 1 is certainly met if ${{\mathsf {a}}}_i\in {{\mathbb {R}}}^m_+$ and $b_i >0$ for at least one $i{ \in \! [{1}\! : \! {m}]}$, taking ${{\mathsf {y}}}= \frac{1}{b_i}{{\mathsf {e}}}_i\in {{\mathbb {R}}}^m_+$.

It is clear that the constraints ${{\mathsf {A}}}{{\mathsf {x}}}={{\mathsf {b}}}$ imply ${{\mathsf {d}}}^\top {{\mathsf {x}}}={{\mathsf {y}}}^\top {{\mathsf {b}}}= 1$. From the decomposition (8) and as in the proof of Theorem 4 we have ${{\mathsf {d}}}^\top {{\mathsf {z}}}_k = {{\mathsf {y}}}^\top {{\mathsf {A}}}{{\mathsf {z}}}_k = {{\mathsf {y}}}^\top {{\mathsf {o}}}= 0$ for all $k\in K_0$ and

$$\begin{aligned} {{\mathsf {x}}}= \sum _{k\in K_+}\lambda _k{{\mathsf {z}}}_k = \sum _{k\in K_+}\lambda _k^2{{\mathsf {x}}}_k \end{aligned}$$

with ${{\mathsf {A}}}{{\mathsf {x}}}_k={{\mathsf {b}}}$, entailing ${{\mathsf {d}}}^\top {{\mathsf {x}}}_k=1$ for all $k\in K_+$, yielding

$$\begin{aligned} {{\mathsf {X}}}{{\mathsf {d}}}= \sum _{k\in K}{{\mathsf {z}}}_k {{\mathsf {z}}}_k^\top {{\mathsf {d}}}= \sum _{k\in K_+}\lambda _k^2\, {{\mathsf {x}}}_k{{\mathsf {x}}}_k^\top {{\mathsf {d}}}+ \sum _{k\in K_0}{{\mathsf {z}}}_k {{\mathsf {z}}}_k^\top {{\mathsf {d}}}= \sum _{k\in K_+}\lambda _k^2\, {{\mathsf {x}}}_k + {{\mathsf {o}}}= {{\mathsf {x}}}\, , \end{aligned}$$

which allows us to rewrite (C) as:

$$\begin{aligned} \begin{aligned} \inf \quad&\langle {{{\mathsf {Q}}}+ ({{\mathsf {d}}}{{\mathsf {c}}}^\top + {{\mathsf {c}}}{{\mathsf {d}}}^\top )} , {{{\mathsf {X}}}} \rangle = \langle {{{\mathsf {Q}}}} , {{{\mathsf {X}}}} \rangle + 2{{\mathsf {c}}}^\top {{\mathsf {X}}}{{\mathsf {d}}}\\ s.t. \quad&\langle { {{\mathsf {d}}}{{\mathsf {a}}}_i^\top + {{\mathsf {a}}}_i{{\mathsf {d}}}^\top } , {{{\mathsf {X}}}} \rangle =2{{\mathsf {a}}}_{i}^{\top }{{\mathsf {X}}}{{\mathsf {d}}}= 2b_{i}\, , \quad i{ \in \! [{1}\! : \! {m}]}\, ,\\&\langle {{{\mathsf {a}}}_i {{\mathsf {a}}}_i^\top } , {{{\mathsf {X}}}} \rangle = {{\mathsf {a}}}_{i}^\top {{\mathsf {X}}} {{\mathsf {a}}}_{i} = b^{2}_{i}\, , \quad i{ \in \! [{1}\! : \! {m}]}\, ,\\&\langle {{{\mathsf {F}}}} , {{{\mathsf {X}}}} \rangle =0\, ,\\&{{\mathsf {X}}}\in {\mathcal{CP}\mathcal{}}^{n}\, . \end{aligned}\quad \qquad \qquad \qquad ({\text {C}_{sim}}) \end{aligned}$$

We abbreviate ${{\mathsf {G}}}:={{\mathsf {Q}}}+ ({{\mathsf {d}}}{{\mathsf {c}}}^\top + {{\mathsf {c}}}{{\mathsf {d}}}^\top )$ and ${{\mathsf {A}}}_i := \frac{1}{2}({{\mathsf {d}}}{{\mathsf {a}}}_i^\top + {{\mathsf {a}}}_i{{\mathsf {d}}}^\top )$ as well as ${\bar{{{\mathsf {A}}}}}_{i}:= {{\mathsf {a}}}_i{{\mathsf {a}}}_i^\top$ for all $i{ \in \! [{1}\! : \! {m}]}$ to rewrite (${\text {C}_{sim}}$) in a simpler form, recalling that $\langle {{{\mathsf {F}}}} , {{{\mathsf {X}}}} \rangle = 0 \Leftrightarrow \langle {{{\mathsf {E}}}} , {{{\mathsf {X}}}} \rangle = 0$:

CP $$\begin{aligned} \begin{aligned} \inf \quad&\langle {{{\mathsf {G}}}} , {{{\mathsf {X}}}} \rangle \\ s.t. \quad&\langle {{{\mathsf {A}}}_i} , {{{\mathsf {X}}}} \rangle = b_{i}\, , \quad i{ \in \! [{1}\! : \! {m}]}\, ,\\&\langle {{\bar{{{\mathsf {A}}}}}_i} , {{{\mathsf {X}}}} \rangle = b^{2}_{i}\, , \quad i{ \in \! [{1}\! : \! {m}]}\, ,\\&\langle {{{\mathsf {E}}}} , {{{\mathsf {X}}}} \rangle = 0\, ,\\&{{\mathsf {X}}}\in {\mathcal{CP}\mathcal{}}^{n}\, .\\ \end{aligned} \end{aligned}$$

By introducing dual variables ${{\mathsf {r}}}\in {\mathbb {R}}^{m}$ for constraints $\langle {{{\mathsf {A}}}_i} , {{{\mathsf {X}}}} \rangle = b_{i}$, ${{\mathsf {t}}}\in {\mathbb {R}}^{m}$ for constraints $\langle {{\bar{{{\mathsf {A}}}}}_i} , {{{\mathsf {X}}}} \rangle = b_{i}^2$ and $h\in {\mathbb {R}}$ for the constraint $\langle {{{\mathsf {E}}}} , {{{\mathsf {X}}}} \rangle = 0$, the dual problem of  (CP) can be obtained:

CD $$\begin{aligned} \begin{aligned} \sup \quad&\sum _{i=1}^{m}(r_{i}b_{i}+t_{i}b_i^2) \\ s.t. \quad&{{\mathsf {S}}}({{\mathsf {r}}},{{\mathsf {t}}},h):= {{\mathsf {G}}}- \sum _{i=1}^{m}(r_i{{\mathsf {A}}}_i+t_i{\bar{{{\mathsf {A}}}}}_i)-h{{\mathsf {E}}}\in \mathcal {COP}^{n}\\&({{\mathsf {r}}},{{\mathsf {t}}},h)\in {\mathbb {R}}^{m}\times {\mathbb {R}}^{m}\times {\mathbb {R}}\, .\\ \end{aligned} \end{aligned}$$

While we have, by preceding arguments under the assumption of $(L_\infty \cap F )$-copositivity of ${{\mathsf {Q}}}$ and $z_P^*\in {{\mathbb {R}}}$,

$$\begin{aligned} z_P^*=z_C^*= \inf \left\{ \langle {{{\mathsf {G}}}} , {{{\mathsf {X}}}} \rangle : {{\mathsf {X}}} \text{ is } \,(\hbox {CP})\text{-feasible }\right\} \end{aligned}$$

as the optimal value for (CP), we denote by

$$\begin{aligned} z_D^*:=\sup \left\{ \sum _{i=1}^{m}(r_{i}b_{i}+t_{i}b_i^2) : {{\mathsf {S}}}({{\mathsf {r}}},{{\mathsf {t}}},h)\in \mathcal {COP}^{n} \right\} \end{aligned}$$

the optimal value for (CD).

We will now establish sufficient conditions for strict feasibility of (CD), which means that Slater’s condition holds for (CD). The condition seems quite natural as it is a sharpening of the conclusion of Lemma 2. It is met if ${{\mathsf {Q}}}$ is positive-definite, and for general ${{\mathsf {Q}}}$ if *L* is bounded (implying $L_\infty = \left\{ {{\mathsf {o}}}\right\}$), by default:

#### Theorem 5

Suppose that $z_P^*\in {{\mathbb {R}}}$ and that ${{\mathsf {Q}}}$ is strictly $L_\infty$-copositive: ${{\mathsf {z}}}^\top {{\mathsf {Q}}} {{\mathsf {z}}}> 0$ if ${{\mathsf {z}}}\in L_\infty \setminus \left\{ {{\mathsf {o}}}\right\}$. Then

$$\begin{aligned} z_C^*=z_D^* \quad \text{ and } \text{ the } \text{ optimal } \text{ value } z_C^* \text{ is } \text{ attained. } \end{aligned}$$

#### Proof

First observe that by definition of ${{\mathsf {d}}}= {{\mathsf {A}}}^\top {{\mathsf {y}}}$, we have ${{\mathsf {z}}}^\top {{\mathsf {d}}}= ({{\mathsf {A}}}{{\mathsf {z}}})^\top {{\mathsf {d}}}= 0$ for all ${{\mathsf {z}}}\in L_\infty$, hence ${{\mathsf {z}}}^\top {{\mathsf {G}}} {{\mathsf {z}}}= {{\mathsf {z}}}^\top {{\mathsf {Q}}} {{\mathsf {z}}}$ on $L_\infty$. Now, under the assumptions, there is a constant $\sigma >0$ such that

$$\begin{aligned} {{\mathsf {z}}}^\top {{\mathsf {G}}} {{\mathsf {z}}}= {{\mathsf {z}}}^\top {{\mathsf {Q}}} {{\mathsf {z}}}\ge 2\sigma \quad \text{ for } \text{ all } {{\mathsf {z}}}\in \Delta _\infty := L_\infty \cap \Delta ^n \,. \end{aligned}$$

By continuity, there is a $\delta >0$ such that also in a small relative neighbourhood

$$\begin{aligned} U_\delta :=\left\{ {{\mathsf {x}}}\in \Delta ^n : {\mathrm { dist}}\,({{\mathsf {x}}}, \Delta _\infty ) < \delta \right\} \end{aligned}$$

we have ${{\mathsf {z}}}^\top {{\mathsf {G}}} {{\mathsf {z}}}\ge \sigma >0$. The continuous function ${ \Vert {{\mathsf {A}}}{{\mathsf {x}}} \Vert }^2$ takes its positive minimum $\mu >0$ on the compact set $\Delta ^n\setminus U_\delta$ which is disjoint from $L_\infty$; furthermore, we have that $\gamma := \min \limits _{{{\mathsf {x}}}\in \Delta ^n} {{\mathsf {z}}}^\top {{\mathsf {G}}} {{\mathsf {z}}}\in {{\mathbb {R}}}$. Choosing $\alpha := \max \left\{ 0,\frac{\sigma -\gamma }{\mu }\right\}$ gives therefore a strictly copositive matrix ${{\mathsf {S}}}({{\mathsf {o}}},-\alpha \,{{\mathsf {e}}},0)$ with a quadratic form ${{\mathsf {z}}}^\top {{\mathsf {G}}} {{\mathsf {z}}}+ \alpha \,{ \Vert {{\mathsf {A}}}{{\mathsf {z}}} \Vert }^2$ taking values not smaller than $\sigma$ over $U_\delta$ but also, by choice of $\alpha$, not smaller than $\sigma$ over $\Delta ^n\setminus U_\delta$. Hence Slater’s condition holds for (CD) and the claim follows. $\square$

## QPCC and conic formulations for quadratic problems under sparsity constraints

In this section, we discuss two models for pursuing sparsity in quadratic optimization problems with the direct use of $\Vert \cdot \Vert _{0}$ put forward by [14, 25]. Both are based upon the following idea: negating the binary variables $|\text{ sign } (y_i)|\in \left\{ 0,1\right\}$, we arrive again at binary variables $u_i:= 1-|\text{ sign } (y_i)|\in \left\{ 0,1\right\}$ which satisfy $u_i|y_i|=0$ for all *i*. As no negative variables appear in this complementarity relation, we can proceed towards a QPCC with a single homogeneous bilinear equality constraint. An additional interesting feature of this approach is that binarity of $u_i$ is not needed here explicitly as a constraint, only the upper bounds $u_i \le 1$ are already sufficient, as will be argued below. As already noted in the introduction, a naive approach via Burer’s result [15] would result in additional constraints in the conic formulation which may be detrimental to algorithmic performance. Anyhow, some of them may be added in the implementation of specially structured problems.

### Soft sparsity: regularization by zero-norm

#### No sign constraints on the variables

We will first treat sparse quadratic problems under linear equality constraints, but no sign constraints on the variables. At first sight, this may seem a restriction of models but actually any sign constraint on some variables would reduce dimensionality rather than increase complexity, as will be clear in the next subsection.

So consider the following soft sparsity model:

$$\begin{aligned} \min _{{{\mathsf {y}}}\in {\mathbb {R}}^n} \left\{ {{\mathsf {y}}}^\top {{\mathsf {Q}}}_{0} {{\mathsf {y}}} + 2{{\mathsf {c}}}_{0}^\top {{\mathsf {y}}}+ \rho \Vert {{\mathsf {y}}}\Vert _0 :\, {{\mathsf {c}}}_i^\top {{\mathsf {y}}}=b_i\, , i{ \in \! [{1}\! : \! {m}]}\right\} \, , \quad \qquad \qquad \qquad ({\text {P}_{S_0}}) \end{aligned}$$

where $\rho >0$ is a regularization parameter for balancing accuracy against sparsity: the larger $\rho$, the more sparse the solution obtained from ((${\text {P}_{S_0}}$)) will be.

By introduction of new variables ${{\mathsf {u}}}\in {\mathbb {R}}^{n}_{+}$ and decomposition of ${{\mathsf {y}}}={{\mathsf {v}}}-{{\mathsf {w}}}$, where $\left\{ {{\mathsf {v}}},{{\mathsf {w}}}\right\} \subset {\mathbb {R}}^{n}_{+}$, problem ((${\text {P}_{S_0}}$)) can be exactly expressed by a quadratic problem:

$$\begin{aligned} \begin{aligned} \min _{\left\{ {{\mathsf {v}}},{{\mathsf {w}}},{{\mathsf {u}}}\right\} \subset {\mathbb {R}}^n_+} \quad&({{\mathsf {v}}}-{{\mathsf {w}}})^\top {{\mathsf {Q}}}_{0} ({{\mathsf {v}}}-{{\mathsf {w}}})+2{{\mathsf {c}}}_{0}^\top ({{\mathsf {v}}}-{{\mathsf {w}}}) + \rho n- \rho \, {{\mathsf {e}}}^\top {{\mathsf {u}}}\\ s.t. \quad&{{\mathsf {c}}}_i ^\top ({{\mathsf {v}}}-{{\mathsf {w}}})=b_i\, ,\quad i{ \in \! [{1}\! : \! {m}]}\, ,\\&{{\mathsf {u}}}^\top ({{\mathsf {v}}}+{{\mathsf {w}}})=0\, ,\\&{{\mathsf {u}}}\le {{\mathsf {e}}}\, . \end{aligned}\quad \qquad \qquad \qquad ({\text {P}_{S^{'}}}) \end{aligned}$$

Because of nonnegativity of $({{\mathsf {u}}},{{\mathsf {v}}},{{\mathsf {w}}})$, the homogeneous bilinear equality constraint ${{\mathsf {u}}}^\top ({{\mathsf {v}}}+{{\mathsf {w}}})=0$ is equivalent to the equality system $u_i(v_{i}+w_{i})=0$, $i{ \in \! [{1}\! : \! {n}]}$.

##### Theorem 6

Problems (${\text {P}_{S_0}}$) and  (${\text {P}_{S^{'}}}$) are equivalent.

##### Proof

This was proved in [25], but we provide a short argument for the readers’ convenience here. For any $\alpha \in {{\mathbb {R}}}$, let $\alpha _+:=\max \left\{ \alpha , 0\right\}$ and $\alpha _- = \alpha _+-\alpha$; then $\alpha _\pm \ge 0$ and $|\alpha |= \alpha _++\alpha _-$ as well as $\alpha = \alpha _+-\alpha _-$. If ${{\mathsf {y}}}$ is feasible to (${\text {P}_{S_0}}$), then putting $v_i=(y_i)_+$ and $w_i = (y_i)_-$ as well as $u_i = 1-|\text{ sign } y_i|\in \left\{ 0,1\right\}$ one gets a feasible solution to (${\text {P}_{S^{'}}}$) with same objective value, so $opt(\text {P}_{S'})\le opt(\text {P}_{S_0})$. Conversely, let

$$\begin{aligned} F':=\{({{\mathsf {v}}},{{\mathsf {w}}},{{\mathsf {u}}})\in {\mathbb {R}}^{3n}_{+} : {{\mathsf {u}}}^\top ({{\mathsf {v}}}+{{\mathsf {w}}})=0, \ {{\mathsf {u}}}\le {{\mathsf {e}}}\, \}\, . \end{aligned}$$

For $({{\mathsf {v}}},{{\mathsf {w}}},{{\mathsf {u}}})\in F'$ feasible to (${\text {P}_{S^{'}}}$), put ${{\mathsf {y}}}:= {{\mathsf {v}}}-{{\mathsf {w}}}\in {{\mathbb {R}}}^n$ and consider $v_i':=(y_i)_+$, $w_i':=(y_i)_-$ and $u_i':=\text{ sign } u_i$. Then also $({{\mathsf {v}}}',{{\mathsf {w}}}',{{\mathsf {u}}}')\in F'$ is (${\text {P}_{S^{'}}}$)-feasible with lower or equal objective value due to ${{\mathsf {e}}}^\top {{\mathsf {u}}}\le {{\mathsf {e}}}^\top {{\mathsf {u}}}'$ and ${{\mathsf {v}}}'-{{\mathsf {w}}}'={{\mathsf {v}}}-{{\mathsf {w}}}$, and again, this value coincides with the objective value of ${{\mathsf {y}}}$ in (${\text {P}_{S_0}}$). Thus $opt(\text {P}_{S_0}) \le opt(\text {P}_{S'})$. $\square$

Given $({{\mathsf {v}}},{{\mathsf {w}}},{{\mathsf {u}}})\in F'$, we introduce slack variables ${{\mathsf {s}}}:={{\mathsf {e}}}-{{\mathsf {u}}}\in {{\mathbb {R}}}^n_+$ if ${{\mathsf {u}}}\le {{\mathsf {e}}}$ and stack all variables to ${{\mathsf {x}}}:=[{{\mathsf {v}}}^{\top },{{\mathsf {w}}}^{\top },{{\mathsf {u}}}^{\top }, {{\mathsf {s}}}^\top ]^{\top }\in {\mathbb {R}}^{4n}_+$. Then problem (${\text {P}_{S^{'}}}$) can be rewritten, dropping the additive constant $\rho n$:

$$\begin{aligned} \begin{aligned} \min _{{{\mathsf {x}}}\in {\mathbb {R}}_{+}^{4n}} \quad&{{\mathsf {x}}}^\top {{\mathsf {Q}}} {{\mathsf {x}}}+ 2{{\mathsf {c}}}^{\top }{{\mathsf {x}}}\\ s.t. \quad&{{\mathsf {a}}}_i^{\top }{{\mathsf {x}}}=b_i\, ,\quad i{ \in \! [{1}\! : \! {m+n}]}\, ,\\&\sum _{(i,j)\in {\mathcal {E}}}x_{i}x_{j}=0\, , \end{aligned}\quad \qquad \qquad \qquad ({\text {P}_{S}}) \end{aligned}$$

where

$$\begin{aligned} {{\mathsf {Q}}}= \begin{pmatrix} {{\mathsf {Q}}}_{0} &{} -{{\mathsf {Q}}}_0 &{} {{\mathsf {O}}}\\ -{{\mathsf {Q}}}_0 &{} {{\mathsf {Q}}}_{0} &{} {{\mathsf {O}}}\\ {{\mathsf {O}}}&{} {{\mathsf {O}}}&{} {{\mathsf {O}}}\\ \end{pmatrix} \in {{\mathcal {S}}}^{4n}\, \text{ and } \quad {{\mathsf {c}}}=[{{\mathsf {c}}}_0^{\top },-{{\mathsf {c}}}_0^{\top },-\frac{\rho }{2}{{\mathsf {e}}}^{\top }, {{\mathsf {o}}}^\top ]^\top \in {\mathbb {R}}^{4n}\, , \end{aligned}$$

as well as the linear constraints, putting $b_i = 1$ for all $i{ \in \! [{m+1}\! : \! {m+n}]}$ while keeping all $b_i$ unchanged for $i{ \in \! [{1}\! : \! {m}]}$:

$$\begin{aligned} {{\mathsf {a}}}_i={\left\{ \begin{array}{ll} [{{\mathsf {c}}}_{i}^{\top },-{{\mathsf {c}}}_{i}^{\top },{{\mathsf {o}}}^{\top }]^{\top }\in {\mathbb {R}}^{4n}\, , &{}\quad \text{ if } i{ \in \! [{1}\! : \! {m}]}\, ,\\ {{\mathsf {e}}}_{i-m+2n} + {{\mathsf {e}}}_{i-m+3n}\in {\mathbb {R}}^{4n}\, , &{}\quad \text{ if } i{ \in \! [{m+1}\! : \! {m+n}]}\, . \end{array}\right. } \end{aligned}$$

Furthermore, we use the graph ${\mathcal {G}}_{{\mathsf {F}}}= (V,{\mathcal {E}})$ with $V=[1\!:\! 4n]$ and edge set ${\mathcal {E}}=\left\{ \left\{ i+2n,j\right\} :i{ \in \! [{1}\! : \! {n}]}, j\in \left\{ i,i+n\right\} \right\}$ to express the bilinear constraints ${{\mathsf {u}}}^\top {{\mathsf {v}}}+ {{\mathsf {u}}}^\top {{\mathsf {w}}}=0$ modelling $u_i|y_i|=0$.

The first 2*n* variables $({{\mathsf {v}}},{{\mathsf {w}}})$ are not necessarily bounded but nevertheless are involved in the complementarity constraint ${{\mathsf {u}}}^\top ({{\mathsf {v}}}+{{\mathsf {w}}}) = 0$, so the classical key condition (K) fails. Fortunately, by construction we find a vertex cover $C:=[2n+1\!:\!3n]$ of ${{\mathcal {G}}}_{{\mathsf {F}}}$ such that any $x_j =u_{j-2n}\le 1$ for all $j\in C$. Moreover, for any feasible solution ${{\hat{{{\mathsf {y}}}}}}\in {{\mathbb {R}}}^n$ to the original QP (i.e., such that ${{\mathsf {C}}}{{\hat{{{\mathsf {y}}}}}}= {{\mathsf {b}}}$),

$$\begin{aligned} {{\hat{{{\mathsf {x}}}}}} ^\top = [({\hat{y}}_1)^+,\ldots , ({\hat{y}}_n)^+,({\hat{y}}_1)^-,\ldots , ({\hat{y}}_n)^-, {{\mathsf {o}}}^\top , {{\mathsf {e}}}^\top ] \end{aligned}$$

satisfies ${{\hat{{{\mathsf {x}}}}}}_C = {{\mathsf {o}}}$, so that condition (R) is met if the original QP is feasible. Lemma 2 can be applied, and yields $L_\infty \subseteq F$ for this class of problems. Note that [3, Thm.4] requires *a priori knowledge* of copositivity of the Hessian of the objective while our approach yields it *automatically* only by property of the constraints alone, and the fact that the optimal objective value is finite.

Therefore the conic relaxation is exact whenever the QP is feasible and bounded, which automatically would be the case for positive-semidefinite ${{\mathsf {Q}}}_0$, given that the linear system ${{\mathsf {c}}}_i^\top {{\mathsf {y}}}=b_i$, $i{ \in \! [{1}\! : \! {m}]}$, has a solution. Furthermore, the slackness constraints ${{\mathsf {u}}}+{{\mathsf {s}}}={{\mathsf {e}}}$ provide some rows ${{\mathsf {a}}}_i\in {{\mathbb {R}}}^{4n}_+$ with $b_i=1>0$, so that also the reduction step as in Sect. 2.3 can be performed.

Unfortunately, the constructed ${{\mathsf {Q}}}$ is never strictly $L_\infty$-copositive even if ${{\mathsf {Q}}}_0$ is positive-definite, hence we cannot establish strong duality along the general lines in Sect. 2.3. Note that in the present case, by construction of ${{\mathsf {A}}}$,

14 $$\begin{aligned} L_\infty = \left\{ [{{\mathsf {v}}}^\top ,{{\mathsf {w}}}^\top , {{\mathsf {o}}}^\top ]^\top \in {{\mathbb {R}}}^{4n}_+ : {{\mathsf {c}}}_i^\top ({{\mathsf {v}}}-{{\mathsf {w}}}) = 0\, ,\, i{ \in \! [{1}\! : \! {m}]}\right\} \, , \end{aligned}$$

which includes all vectors of the form $[{{\mathsf {v}}}^\top ,{{\mathsf {v}}}^\top , {{\mathsf {o}}}^\top ]^\top \in {{\mathbb {R}}}^{4n}_+$ for which any slack matrix ${{\mathsf {S}}}({{\mathsf {r}}},{{\mathsf {t}}},h)$ gives a zero quadratic form.

However, even if ${{\mathsf {Q}}}_0$ is merely positive-semidefinite, adding an $\ell ^2$ term $\tau \sum \limits _{i=1}^n (v_i^2+w_i^2)$ with small $\tau >0$ to the objective provides a regularization which ensures strong conic duality. See Sect. 3.3 for more details and Sect. 5 for the effect of $\tau$.

#### Sparsity in QPs with sign constraints on variables

Additional linear inequality constraints can also be incorporated by introducing additional slack variables. We leave the obvious adaptations to the interested readers and concentrate for ease of presentation on the following model:

$$\begin{aligned} \min _{{{\mathsf {y}}}\in {\mathbb {R}}^n_+} \left\{ {{\mathsf {y}}}^\top {{\mathsf {Q}}}_{0} {{\mathsf {y}}} + 2{{\mathsf {c}}}_{0}^\top {{\mathsf {y}}}+ \rho \Vert {{\mathsf {y}}}\Vert _0 :\, {{\mathsf {c}}}_i^\top {{\mathsf {y}}}=b_i\, , i{ \in \! [{1}\! : \! {m}]}\right\} \, .\quad \qquad \qquad \qquad ({\text {P}_{S}^+}) \end{aligned}$$

Here we need not split ${{\mathsf {y}}}={{\mathsf {v}}}-{{\mathsf {w}}}$ into two nonnegative vectors any more, but we will keep ${{\mathsf {u}}}$ and the slack variables as in the previous subsection. So, here ${{\mathsf {x}}}^\top = [{{\mathsf {y}}}^\top , {{\mathsf {u}}}^\top , {{\mathsf {s}}}^\top ]$ with ${{\mathsf {x}}}\in {{\mathbb {R}}}^{3n}_+$ now, and we end up with a QPCC formulation very similar to (${\text {P}_{S}}$), but with reduced dimensionality 3*n* and smaller primitive data: ${{\mathsf {x}}}^\top {{\mathsf {Q}}} {{\mathsf {x}}}+ 2{{\mathsf {c}}}^\top {{\mathsf {x}}}= {{\mathsf {y}}}^\top {{\mathsf {Q}}}_0 {{\mathsf {y}}} + 2{{\mathsf {c}}}_0^\top {{\mathsf {y}}}$ for

$$\begin{aligned} {{\mathsf {Q}}}= \begin{pmatrix} {{\mathsf {Q}}}_{0} &{} {{\mathsf {O}}}\\ {{\mathsf {O}}}&{} {{\mathsf {O}}}\\ \end{pmatrix} \in {{\mathcal {S}}}^{3n}\, \text{ and } \quad {{\mathsf {c}}}=[{{\mathsf {c}}}_0^{\top },-\frac{\rho }{2}{{\mathsf {e}}}^{\top }, {{\mathsf {o}}}^\top ]^\top \in {\mathbb {R}}^{3n}\, , \end{aligned}$$

The linear constraints read now, putting $b_i = 1$ for all $i{ \in \! [{m+1}\! : \! {m+n}]}$ while keeping $b_i$ unchanged for all $i{ \in \! [{1}\! : \! {m}]}$:

$$\begin{aligned} {{\mathsf {a}}}_i={\left\{ \begin{array}{ll} [{{\mathsf {c}}}_{i}^{\top },{{\mathsf {o}}}^{\top }]^{\top }\in {\mathbb {R}}^{3n}\, , &{}\quad \text{ if } i{ \in \! [{1}\! : \! {m}]}\, ,\\ {{\mathsf {e}}}_{i-m+n} + {{\mathsf {e}}}_{i-m+2n}\in {\mathbb {R}}^{3n}\, , &{}\quad \text{ if } i{ \in \! [{m+1}\! : \! {m+n}]}\, .\end{array}\right. } \end{aligned}$$

Furthermore, we use the edge set ${\mathcal {E}}=\left\{ \left\{ i,i+n\right\} :i{ \in \! [{1}\! : \! {n}]}\right\}$ to express the bilinear constraints ${{\mathsf {u}}}^\top {{\mathsf {y}}}=0$ modelling $u_iy_i=0$. Here $C = [n+1\!:\!2n]$ is a vertex cover for ${{\mathcal {G}}}_{{\mathsf {F}}}$. Again, similar to the case with no sign constraints, Lemma 2 can be applied and yields $L_{\infty } \subseteq F$ also for this problem class. Indeed, for any feasible ${{\hat{{{\mathsf {y}}}}}}$ here ${{\hat{{{\mathsf {x}}}}}} := [{{\hat{{{\mathsf {y}}}}}}, {{\mathsf {o}}}^\top , {{\mathsf {e}}}^\top ]^\top \in L$ satisfies ${{\hat{{{\mathsf {x}}}}}}_C = {{\mathsf {o}}}$.

The two resulting, already simplified conic reformulation problems read exactly as in (CP) and (CD) for their duals, with *n* replaced by 3*n* and *m* replaced by $m+n$.

Apart from employing a conic representation with reduced dimensionality, we also can infer strong duality for the conic primal-dual pair by imposing conditions which in a realistic setting are met more frequently, compared to the setting with no sign constraints on the primitive variables. Indeed, as explained in the remark after the following proof, if the primitive polyhedron is bounded, then the strict copositivity condition holds by default.

##### Theorem 7

In model ($\text {P}_{S}^+$), assume that ${{\mathsf {Q}}}_0$ is strictly $L_\infty ^{{\mathsf {C}}}$-copositive with

$$\begin{aligned} L_\infty ^{{\mathsf {C}}}:=\left\{ {{\mathsf {y}}}\in {{\mathbb {R}}}^n_+ : {{\mathsf {C}}}{{\mathsf {y}}}={{\mathsf {o}}}\right\} \quad \text{ where } {{\mathsf {C}}}^\top = [{{\mathsf {c}}}_1,\ldots , {{\mathsf {c}}}_m]\, , \end{aligned}$$

i.e., ${{\mathsf {y}}}^\top {{\mathsf {Q}}}_0 {{\mathsf {y}}} > 0$ for all ${{\mathsf {y}}}\in {{\mathbb {R}}}^n_+\setminus \left\{ {{\mathsf {o}}}\right\}$ with ${{\mathsf {C}}}{{\mathsf {y}}}={{\mathsf {o}}}$. Then

- any feasible problem instance is bounded below;
- the conic representation is exact;
- the primal-dual conic pair has zero duality gap.

##### Proof

Observe that in our case $L_\infty = L_\infty ^{{\mathsf {C}}}\times \left\{ {{\mathsf {o}}}\right\} \subset {{\mathbb {R}}}^{3n}_+$, so that ${{\mathsf {z}}}\in L_\infty \setminus \left\{ {{\mathsf {o}}}\right\}$ if and only if $z^\top = [{{\mathsf {y}}}^\top ,{{\mathsf {o}}}^\top ]$ with ${{\mathsf {y}}}\in L_\infty ^{{\mathsf {C}}}\setminus \left\{ {{\mathsf {o}}}\right\}$, and then ${{\mathsf {z}}}^\top {{\mathsf {Q}}} {{\mathsf {z}}}= {{\mathsf {y}}}^\top {{\mathsf {Q}}}_0 {{\mathsf {y}}} > 0$ by assumption. The claims now follows by application of Theorem 5. $\square$

Note that the assumption that ${{\mathsf {Q}}}_0$ be strictly $L_\infty ^{{\mathsf {C}}}$-copositive is trivially satisfied if the primitive feasible polyhedron $M=\left\{ {{\mathsf {y}}}\in {{\mathbb {R}}}^n_+: {{\mathsf {C}}}{{\mathsf {y}}}={{\mathsf {b}}}\right\}$ is bounded, because then $L_\infty ^{{\mathsf {C}}}=\left\{ {{\mathsf {o}}}\right\}$.

### Hard sparsity constraints

Sometimes we want to force the solution of a quadratic problem to be *k*-sparse, where the integer $k>0$ is a hard parameter for sparsity. To this end, we consider another sparsity model for constrained quadratic problems:

$$\begin{aligned} \min _{{{\mathsf {y}}}\in {\mathbb {R}}^n} \left\{ {{\mathsf {y}}}^\top {{\mathsf {Q}}}_{0} {{\mathsf {y}}} + 2{{\mathsf {c}}}_{0}^\top {{\mathsf {y}}}:\, {{\mathsf {c}}}_i^\top {{\mathsf {y}}}=b_i\, , i{ \in \! [{1}\! : \! {m}]}\, ,\quad \Vert {{\mathsf {y}}}\Vert _0\le k \right\} \, .\quad \qquad \qquad \qquad ({\text {P}_{H_0}}) \end{aligned}$$

Instead of using the soft parameter $\rho$ to pursuit sparsity, alternatively, we employ $\Vert \cdot \Vert _0$ directly in a hard constraint. In analogy to the formulation of the soft sparsity problem (${\text {P}_{S_0}}$), we can also exactly express the hard sparsity problem ($\text {P}_{H_0}$) as follows:

$$\begin{aligned} \begin{aligned} \min _{\left\{ {{\mathsf {v}}},{{\mathsf {w}}},{{\mathsf {u}}}\right\} \subset {\mathbb {R}}^n_+} \quad&({{\mathsf {v}}}-{{\mathsf {w}}})^\top {{\mathsf {Q}}}_{0} ({{\mathsf {v}}}-{{\mathsf {w}}})+2{{\mathsf {c}}}_{0}^\top ({{\mathsf {v}}}-{{\mathsf {w}}}) \\ s.t. \quad&{{\mathsf {c}}}_i ^\top ({{\mathsf {v}}}-{{\mathsf {w}}})=b_i\, ,\quad i{ \in \! [{1}\! : \! {m}]}\, ,\\&{{\mathsf {u}}}^\top ({{\mathsf {v}}}+{{\mathsf {w}}})=0\, ,\\&-{{\mathsf {e}}}^\top {{\mathsf {u}}}\le k-n\, ,\\&{{\mathsf {u}}}\le {{\mathsf {e}}}\, . \end{aligned}\quad \qquad \qquad \qquad ({\text {P}_{H^{'}}}) \end{aligned}$$

#### Theorem 8

Problems ($\text {P}_{H_0}$) and  ($\text {P}_{H^{'}}$) are equivalent.

#### Proof

The proof was presented in [14] and is very similar to that of Theorem 6. $\square$

Similarly, with the help of slack variables ${{\mathsf {s}}}:={{\mathsf {e}}}-{{\mathsf {u}}}\in {{\mathbb {R}}}^n_+$ and $\sigma :={{\mathsf {e}}}^\top {{\mathsf {u}}}+k-n\ge 0$ and after stacking all variables to ${{\mathsf {x}}}:=[{{\mathsf {v}}}^{\top },{{\mathsf {w}}}^{\top },{{\mathsf {u}}}^{\top }, {{\mathsf {s}}}^\top , \sigma ]^{\top }\in {\mathbb {R}}^{4n+1}_+$, the problem ($\text {P}_{H^{'}}$) can be rewritten:

$$\begin{aligned} \begin{aligned} \min _{{{\mathsf {x}}}\in {\mathbb {R}}_{+}^{4n+1}} \quad&{{\mathsf {x}}}^\top {{\mathsf {Q}}} {{\mathsf {x}}}+ 2{{\mathsf {c}}}^{\top }{{\mathsf {x}}}\\ s.t. \quad&{{\mathsf {a}}}_i^{\top }{{\mathsf {x}}}=b_i\, ,\quad i{ \in \! [{1}\! : \! {m+n+1}]}\, ,\\&\sum _{(i,j)\in {\mathcal {E}}}x_{i}x_{j}=0\, , \end{aligned}\quad \qquad \qquad \qquad ({\text {P}_{H}}) \end{aligned}$$

where

$$\begin{aligned} {{\mathsf {Q}}}= \begin{pmatrix} {{\mathsf {Q}}}_{0} &{} -{{\mathsf {Q}}}_0 &{} {{\mathsf {O}}}\\ -{{\mathsf {Q}}}_0 &{} {{\mathsf {Q}}}_{0} &{} {{\mathsf {O}}}\\ {{\mathsf {O}}}&{} {{\mathsf {O}}}&{} {{\mathsf {O}}}\\ \end{pmatrix} \in {{\mathcal {S}}}^{4n+1}\, \text{ and } \quad {{\mathsf {c}}}=[{{\mathsf {c}}}_0^{\top },-{{\mathsf {c}}}_0^{\top }, {{\mathsf {o}}}^\top ]^\top \in {\mathbb {R}}^{4n+1}\, , \end{aligned}$$

as well as the linear constraints, putting $b_i = 1$ for all $i{ \in \! [{m+1}\! : \! {m+n}]}$ and $b_i = k-n$ for $i = m+n+1$:

$$\begin{aligned} {{\mathsf {a}}}_i={\left\{ \begin{array}{ll} {[}{{\mathsf {c}}}_{i}^{\top },-{{\mathsf {c}}}_{i}^{\top },{{\mathsf {o}}}^{\top }]^{\top }\in {\mathbb {R}}^{4n+1}\, , &{}\quad \text{ if } i{ \in \! [{1}\! : \! {m}]}\, ,\\ {{\mathsf {e}}}_{i-m+2n} + {{\mathsf {e}}}_{i-m+3n}\in {\mathbb {R}}^{4n+1}\, , &{}\quad \text{ if } i{ \in \! [{m+1}\! : \! {m+n}]}\, ,\\ {[}{{\mathsf {o}}}^{\top },{{\mathsf {o}}}^{\top },-{{\mathsf {e}}}^{\top },{{\mathsf {o}}}^{\top },1]^{\top }\in {\mathbb {R}}^{4n+1}\, , &{}\quad \text{ if } i=m+n+1\,. \end{array}\right. } \end{aligned}$$

Similarly to the soft sparsity case, the model with sign constraints on the variables can also be treated, leading to

$$\begin{aligned} \min _{{{\mathsf {y}}}\in {\mathbb {R}}^n_+} \left\{ {{\mathsf {y}}}^\top {{\mathsf {Q}}}_{0} {{\mathsf {y}}} + 2{{\mathsf {c}}}_{0}^\top {{\mathsf {y}}}:\, {{\mathsf {c}}}_i^\top {{\mathsf {y}}}=b_i\, , i{ \in \! [{1}\! : \! {m}]}\, ,\quad \Vert {{\mathsf {y}}}\Vert _0\le k \right\} \, .\quad \qquad \qquad \qquad ({\text {P}_{H}^+}) \end{aligned}$$

Again, we need not split ${{\mathsf {y}}}={{\mathsf {v}}}-{{\mathsf {w}}}$ into two nonnegative vectors any more, but we will keep ${{\mathsf {u}}}$ and the slack variables as in the previous subsection. So, here ${{\mathsf {x}}}^\top = [{{\mathsf {y}}}^\top , {{\mathsf {u}}}^\top , {{\mathsf {s}}}^\top , \sigma ]$ with ${{\mathsf {x}}}\in {{\mathbb {R}}}^{3n+1}_+$ now, and again ${{\mathsf {x}}}^\top {{\mathsf {Q}}} {{\mathsf {x}}}+ 2{{\mathsf {c}}}^\top {{\mathsf {x}}}= {{\mathsf {y}}}^\top {{\mathsf {Q}}}_0 {{\mathsf {y}}} + 2{{\mathsf {c}}}_0^\top {{\mathsf {y}}}$. The QPCC formulation is similar to ($\text {P}_{H}$), using the same

$$\begin{aligned} {{\mathsf {Q}}}= \begin{pmatrix} {{\mathsf {Q}}}_{0} &{} {{\mathsf {O}}}\\ {{\mathsf {O}}}&{} {{\mathsf {O}}}\\ \end{pmatrix} \in {{\mathcal {S}}}^{3n+1}\, \text{ and } \quad {{\mathsf {c}}}=[{{\mathsf {c}}}_0^{\top }, {{\mathsf {o}}}^\top ]^\top \in {\mathbb {R}}^{3n+1}\, , \end{aligned}$$

adding a linear constraint with $b_{m+n+1}=k-n$ while keeping $b_i$ unchanged for all $i{ \in \! [{1}\! : \! {m+n}]}$:

$$\begin{aligned} {{\mathsf {a}}}_i={\left\{ \begin{array}{ll} {[}{{\mathsf {c}}}_{i}^{\top },{{\mathsf {o}}}^{\top }]^{\top }\in {\mathbb {R}}^{3n+1}\, , &{}\quad \text{ if } i{ \in \! [{1}\! : \! {m}]}\, ,\\ {{\mathsf {e}}}_{i-m+n} + {{\mathsf {e}}}_{i-m+2n}\in {\mathbb {R}}^{3n+1}\, , &{}\quad \text{ if } i{ \in \! [{m+1}\! : \! {m+n}]}\, \\ {[}{{\mathsf {o}}}^{\top },-{{\mathsf {e}}}^{\top },{{\mathsf {o}}}^{\top },1]^{\top }\in {\mathbb {R}}^{3n+1}\, , &{}\quad \text{ if } i=m+n+1\, .\end{array}\right. } \end{aligned}$$

The conic reformulation problems read exactly as in (CP) and (CD) for their duals, with *n* replaced by ${3n+1}$ and *m* replaced by $m+n+1$.

#### Theorem 9

In model ($\text {P}_{H}^+$), assume that ${{\mathsf {Q}}}_0$ is strictly $L_\infty ^{{\mathsf {C}}}$-copositive with

$$\begin{aligned} L_\infty ^{{\mathsf {C}}}:=\left\{ {{\mathsf {y}}}\in {{\mathbb {R}}}^n_+ : {{\mathsf {C}}}{{\mathsf {y}}}={{\mathsf {o}}}\right\} \quad \text{ where } {{\mathsf {C}}}^\top = [{{\mathsf {c}}}_1,\ldots , {{\mathsf {c}}}_m]\, , \end{aligned}$$

i.e., ${{\mathsf {y}}}^\top {{\mathsf {Q}}}_0 {{\mathsf {y}}} > 0$ for all ${{\mathsf {y}}}\in {{\mathbb {R}}}^n_+\setminus \left\{ {{\mathsf {o}}}\right\}$ with ${{\mathsf {C}}}{{\mathsf {y}}}={{\mathsf {o}}}$. Then

- any feasible problem instance is bounded below;
- the conic representation is exact;
- the primal-dual conic pair has zero duality gap.

#### Proof

Employ the same arguments as for Theorem 7. $\square$

Again, the assumption that ${{\mathsf {Q}}}_0$ be strictly $L_\infty ^{{\mathsf {C}}}$-copositive is trivially satisfied if the primitive feasible polyhedron $M=\left\{ {{\mathsf {y}}}\in {{\mathbb {R}}}^n_+: {{\mathsf {C}}}{{\mathsf {y}}}={{\mathsf {b}}}\right\}$ is bounded, because then $L_\infty ^{{\mathsf {C}}}=\left\{ {{\mathsf {o}}}\right\}$. It is also satisfied if ${{\mathsf {Q}}}_0$ is positive-definite as in the following section which presents an application which motivated this study.

### Sparse linear regression: conic reformulation

In a linear least-squares/regression context, ${{\mathsf {Q}}}_0 = {{\mathsf {A}}}_0^\top {{\mathsf {A}}}_0$ with ${{\mathsf {A}}}_0$ an $m\times n$ matrix with $m<n$, so ${{\mathsf {Q}}}_0$ is always positive-semidefinite but will have some zero eigenvalues. Hence strict copositivity of ${{\mathsf {Q}}}_0$ cannot be ensured by spectral properties of ${{\mathsf {Q}}}_0$ alone; one possibility is the condition that ${{\mathsf {A}}}_0$ contains some strictly positive rows which renders ${{\mathsf {Q}}}_0 = {{\mathsf {P}}}+{{\mathsf {N}}}$ with ${{\mathsf {P}}}\in {{\mathcal {P}}}^n$ and ${{\mathsf {N}}}\in {{\mathcal {N}}}^n$. This is enough to ensure strict ${{\mathbb {R}}}^n_+$-copositivity of ${{\mathsf {Q}}}_0$ which establishes all favorable properties of our conic reformulation by application of Theorems 7 and 9. Another class of instances is that with bounded primitive feasible polyhedron $M=\left\{ {{\mathsf {y}}}\in {{\mathbb {R}}}^n_+: {{\mathsf {C}}}{{\mathsf {y}}}={{\mathsf {b}}}\right\}$, as explained in the note after these results.

In absence of sign constraints, another option already hinted at in Sect. 3.1.1 would consist in adding a regularization term $\tau \sum \limits _{i=1}^n (v_i^2+w_i^2)$, with small $\tau >0$:

#### Proposition 10

Consider a regularized sparse linear regression problem with a small $\tau >0$ where, in the notation of (${\text {P}_{S_0}}$) or ($\text {P}_{H_0}$),

$$\begin{aligned} {{\mathsf {y}}}^\top {{\mathsf {Q}}}_0 {{\mathsf {y}}} = { \Vert {{\mathsf {A}}}_0{{\mathsf {y}}} \Vert }^2 + \tau { \Vert {{\mathsf {y}}} \Vert }^2\, . \end{aligned}$$

Then

- any feasible problem instance is bounded below;
- the conic representation is exact;
- the primal-dual conic pair has zero duality gap.

#### Proof

By reformulation as before, we only need to add $\tau \left( { \Vert {{\mathsf {v}}} \Vert }^2 +{ \Vert {{\mathsf {w}}} \Vert }^2 \right)$ to the original objective of (${\text {P}_{S_0}}$) or ($\text {P}_{H_0}$) instead of $\tau { \Vert {{\mathsf {y}}} \Vert }^2 = \tau { \Vert {{\mathsf {v}}}-{{\mathsf {w}}} \Vert }^2$; indeed, in the optimum we have ${ \Vert {{\mathsf {v}}} \Vert }^2 +{ \Vert {{\mathsf {w}}} \Vert }^2 = { \Vert {{\mathsf {v}}}-{{\mathsf {w}}} \Vert }^2$ as optimality already implies $v_i=y_i^+$ and $w_i=y_i^-$ and therefore ${{\mathsf {v}}}^\top {{\mathsf {w}}}=0$, as argued before. Thus we can regularize by a positive-definite term in $({{\mathsf {v}}},{{\mathsf {w}}})$ which renders ${{\mathsf {Q}}}$ strictly $L_\infty$-copositive where $L_\infty$ is given as in (14) (replace 4*n* with $4n+1$ when dealing with hard sparsity constraints). The assertions then follow by application of Theorems 4 and 5. $\square$

Thus, for a large class of sparse regression problems, be they constrained or not, our theory applies in full strength. Apart from that, even if the last line of arguments cannot be carried through for all instances with no sign constraints on the variables, we still can use weak conic duality to obtain strong dual bounds, as our experiments show.

## An iterative algorithm for dual problems (CD)

For the convenience of expression, we write (CD) in the following form:

15 $$\begin{aligned} \max _{{{\mathsf {x}}}\in {\mathbb {R}}^m} \left\{ {{\mathsf {a}}}^\top {{\mathsf {x}}}: g({{\mathsf {x}}})\in \mathcal {COP}^{n}\right\} \, , \end{aligned}$$

where $g:{\mathbb {R}}^m\rightarrow {\mathcal {S}}^{n}$ is a linear-affine function in ${{\mathsf {x}}}$ and ${{\mathsf {a}}}\in {{\mathbb {R}}}^m$. By convexity of $\mathcal {COP}^{n}$, it is obvious that the set $\{{{\mathsf {x}}}:\, g({{\mathsf {x}}})\in \mathcal {COP}^{n}\}$ is convex too. Therefore it is possible to solve the convex problem (15) via a directional search scheme, provided a feasible initial guess. Recall that any feasible solution to (15) already provides a rigorous lower bound ${{\mathsf {a}}}^\top {{\mathsf {x}}}$ for (P). Now we introduce a derivative-free algorithm for (15) where a subscript *i* denotes the *i*-th entry of a vector and a superscript *k* stands for the iteration counter.
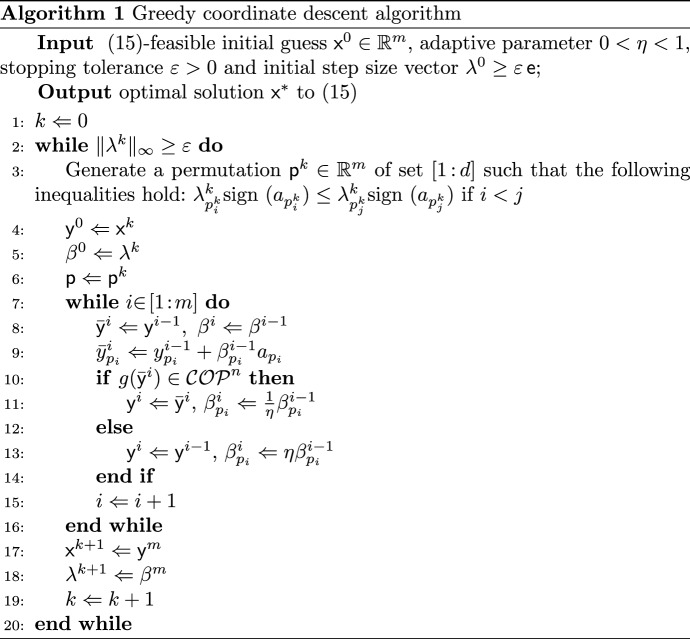


As for the copositivity detection involved in the Algorithm 1, we adopt the method by [2], which amounts to solve the following MILP:

16 $$\begin{aligned} \begin{aligned} \max _{{{\mathsf {x}}},{{\mathsf {y}}}} \quad&\gamma \\ s.t. \quad&{{\mathsf {s}}}_i^\top {{\mathsf {x}}}\le - \gamma + m_i (1- y_i)\, ,\quad i{ \in \! [{1}\! : \! {n}]},\\&\gamma \ge 0\, , {{\mathsf {o}}}\le {{\mathsf {x}}}\le {{\mathsf {y}}},\\&{{\mathsf {y}}}\in \{0,1\}^n\, , {{\mathsf {e}}}^\top {{\mathsf {y}}}\ge 1 \, , \end{aligned} \end{aligned}$$

where ${{\mathsf {s}}}_i$ is the *i*-th column of targeted matrix ${{\mathsf {S}}}$ and the parameter $m_i$ is typically chosen $m_i = 1 + \sum _{i\ne j} \{s_{ij}: s_{ij} >0\}$ as in [2].

### Theorem 11

[2] Let ${{\mathsf {S}}}\in {\mathcal {S}}^{n}$. Then ${{\mathsf {S}}}\in \mathcal {COP}^n$ if and only if the optimal value of (16) is zero.

## Numerical experiments

In this section, we conduct numerical experiments on a repository available online and randomly generated problem sets by adopting the proposed copositive relaxation technique and solving it by Algorithm 1. Moreover, we compare our results with several existing primal approaches in the content of soft and hard regression problems. All the computations are implemented in MATLAB R2021a on a laptop with 3.3Ghz AMD Ryzen 9 processor and 16GB RAM.

### Results on some QPCC instances from MacMPEC

As a first proof of concept for our approach, we take several QPCC instances from the MacMPEC repository [31], namely the larger ones originating from [26] which involve linear constraints arising from network conditions. We bring these instances into the form (P) and report the conic dual bounds of these instances in Table 1, which suggests that our approach provides almost tight bounds for all instances, given that the optimal value is zero, see [31]. Note that the objective functions in these instances are all convex, so that the second implication of Lemma 2 applies and checking (R) is not necessary in this context. The slight increase in the absolute value of dual bounds with increasing problem size may be attributed to the numerical tolerance involved in Algorithm 1.

**Table 1:** Conic dual bounds for some MacMPEC instances

Instance			(*m*, *n*)	Dual bounds	Time (mins.)
flp4-1			(60, 190)	$-0.027$	20.3
flp4-2			(110, 270)	$-0.031$	45.4
flp4-3			(170, 380)	$-0.035$	68.8
flp4-4			(250, 550)	$-0.041$	139.9

The reported computational times show a moderate increase with problem size. For these relatively large instances, they fall into the typical range for conic optimization problems and should be compared to training times for machine learning, including the effort of tuning hyperparameters. Note that most likely, off-the-shelf alternative copositive approaches [15] would not be solvable at all due to memory problems.

### Results on constructed instances

In this subsection we turn to sparse least-square regression models which may take one of the following forms:

17 $$\begin{aligned} \begin{aligned} \min _{{{\mathsf {x}}}\in {\mathbb {R}}^{n}}\quad&F_s({{\mathsf {x}}}) := \frac{1}{2}\Vert {{\mathsf {A}}}_0{{\mathsf {x}}}-{{\mathsf {a}}}_0\Vert ^2+\rho \Vert {{\mathsf {x}}}\Vert _0\\ s.t. \quad&{{\mathsf {B}}}_0{{\mathsf {x}}}= {{\mathsf {b}}}_0\, , \end{aligned} \end{aligned}$$

18 $$\begin{aligned} \begin{aligned} \min _{{{\mathsf {x}}}\in {\mathbb {R}}_{+}^{n}}\quad&F_{sp}({{\mathsf {x}}}) :=\frac{1}{2}\Vert {{\mathsf {A}}}_0{{\mathsf {x}}}-{{\mathsf {a}}}_0\Vert ^2+\rho \Vert {{\mathsf {x}}}\Vert _0\\ s.t. \quad&{{\mathsf {B}}}_0{{\mathsf {x}}}= {{\mathsf {b}}}_0\, , \end{aligned} \end{aligned}$$

19 $$\begin{aligned} \begin{aligned} \min _{{{\mathsf {x}}}\in {\mathbb {R}}^{n}}\quad&F_h({{\mathsf {x}}}) := \frac{1}{2} \Vert {{\mathsf {A}}}_0{{\mathsf {x}}}-{{\mathsf {a}}}_0\Vert ^2\\ s.t. \quad&{{\mathsf {B}}}_0{{\mathsf {x}}}= {{\mathsf {b}}}_0\\&\Vert {{\mathsf {x}}}\Vert _0\le k\, ,\\ \end{aligned} \end{aligned}$$

and

20 $$\begin{aligned} \begin{aligned} \min _{{{\mathsf {x}}}\in {\mathbb {R}}_{+}^{n}} \quad&F_{hp}({{\mathsf {x}}}) := \frac{1}{2} \Vert {{\mathsf {A}}}_0{{\mathsf {x}}}-{{\mathsf {a}}}_0\Vert ^2\\ s.t. \quad&{{\mathsf {B}}}_0{{\mathsf {x}}}= {{\mathsf {b}}}_0\\&\Vert {{\mathsf {x}}}\Vert _0\le k\, ,\\ \end{aligned} \end{aligned}$$

which are special cases of  (${\text {P}_{S_0}}$),  ($\text {P}_{S}^+$),  ($\text {P}_{H_0}$) and  ($\text {P}_{H}^+$) respectively, referred to soft sparse regression problems and hard sparse regression problems. This class of problems actually covers many applications especially in portfolio selection [6, 32] and compressed sensing [4]. It is clear that the Hessians of the quadratic parts in these regression problems are all positive-semidefinite. Alternatively, one here could employ [3, Thm.4] directly for this application, or else employ the branch-and-bound approach in [21].

As discussed in Sect. 3.3, we employ the regularization term $\tau \sum \limits _{i=1}^n (v_i^2+w_i^2)$ to ensure strong conic duality according to Theorem 3.3 and by $R_s$, $R_{sp}$, $R_h$, $R_{hp}$ we denote the objective function of the regularized problems for  (17)–(20) respectively. e.g., the regularized problems for (17):

$$\begin{aligned} \begin{aligned} \min _{\left\{ {{\mathsf {v}}},{{\mathsf {w}}},{{\mathsf {u}}}\right\} \subset {\mathbb {R}}^n_+} \quad&R_s({{\mathsf {x}}}):=\frac{1}{2}({{\mathsf {v}}}-{{\mathsf {w}}})^\top {{\mathsf {Q}}}_{0} ({{\mathsf {v}}}-{{\mathsf {w}}})+{{\mathsf {c}}}_{0}^\top ({{\mathsf {v}}}-{{\mathsf {w}}}) - \rho \, {{\mathsf {e}}}^\top {{\mathsf {u}}}+ \tau \sum \limits _{i=1}^n (v_i^2+w_i^2)\\ s.t. \quad&{{\mathsf {B}}}_0 ({{\mathsf {v}}}-{{\mathsf {w}}}) = {{\mathsf {b}}}_0\\&{{\mathsf {u}}}^\top ({{\mathsf {v}}}+{{\mathsf {w}}})=0\\&{{\mathsf {u}}}\le {{\mathsf {e}}}\\ \end{aligned} \end{aligned}$$

where ${{\mathsf {Q}}}_{0} = {{\mathsf {A}}}_{0}^\top {{\mathsf {A}}}_{0}$ and ${{\mathsf {c}}}_0 = - {{\mathsf {A}}}_{0}^\top {{\mathsf {a}}}_{0}$. In order to reduce the effect of added regularization term, we adopt $\tau = 10^{-5}$ throughout this section.

Following Theorem 4, we then reformulate the above QPCCs as conic problems of the form  (CP) and have its conic dual problems of the form  (CD). Consequently, on the dual side, by using an iterative algorithm for the copositive optimization problems (CD), we obtain conic dual bounds for all sparse regression problems (17)–(20).

#### Instance generation

For soft sparse regression, an instance of the form  (17) or  (18) can be determined by parameters ${{\mathsf {A}}}_0$, ${{\mathsf {a}}}_0$, ${{\mathsf {B}}}_0$, ${{\mathsf {b}}}_0$ and $\rho$. To generate the soft problem set, firstly we generate measurement matrix ${{\mathsf {A}}}_0\in {\mathbb {R}}^{d \times n} (d<n)$ with standard Gaussian entries and $k_0$-sparse vector ${{\mathsf {x}}}_{0}^{*}\in {\mathbb {R}}^{n}$(${{\mathsf {x}}}_{0}^{*}\in {\mathbb {R}}_{+}^{n}$). Then let ${{\mathsf {a}}}_0 := {{\mathsf {A}}}_0{{\mathsf {x}}}_{0}^{*}+0.01\xi \in {\mathbb {R}}^d$ where $\xi$ is standard Gaussian noise. Regarding the choice of ${{\mathsf {B}}}_0$ and ${{\mathsf {b}}}_0$, apart from generating it with standard Gaussian entries, we propose two methods to construct ${{\mathsf {B}}}_0$ such that the Hessian of the objective, ${{\mathsf {A}}}_0^\top {{\mathsf {A}}}_0$, is strictly $L_{\infty }$-copositive: for any ${{\mathsf {x}}}\in L_{\infty }:= \{{{\mathsf {x}}}\in {\mathbb {R}}^{n}_+ : {{\mathsf {B}}}_0 {{\mathsf {x}}}= {{\mathsf {o}}}\}$ we have ${{\mathsf {x}}}^\top {{\mathsf {A}}}_0 ^\top {{\mathsf {A}}}_0{{\mathsf {x}}}> 0$.

- **Choice 1**: First, we define a set $\bar{{\mathfrak {B}}}:= \{{{\mathsf {a}}}_i-\varepsilon {{\mathsf {e}}}\in {\mathbb {R}}^n: i{ \in \! [{1}\! : \! {d}]}, \varepsilon \ne 0\}$, where ${{\mathsf {a}}}_i^\top$ is the *i*-th row of matrix ${{\mathsf {A}}}_0$, and ${{\mathsf {B}}}_0$ can be chosen as any element of the set ${\mathfrak {B}} := \{[{{\mathsf {b}}}_1^\top , {{\mathsf {b}}}_2^\top ,\ldots , {{\mathsf {b}}}_m^\top ]^\top \in {\mathbb {R}}^{m\times n}: {{\mathsf {b}}}_i \in \bar{{\mathfrak {B}}}, i{ \in \! [{1}\! : \! {m}]}\}$. Indeed, if ${{\mathsf {x}}}\ne {{\mathsf {o}}}$ lies in the null space of ${{\mathsf {B}}}_0 \in {\mathfrak {B}}$, then there exists $j { \in \! [{1}\! : \! {m}]}$ such that $({{\mathsf {a}}}_j - \varepsilon {{\mathsf {e}}})^\top {{\mathsf {x}}}= 0$. Now consider
  $$\begin{aligned} {{\mathsf {x}}}^\top {{\mathsf {A}}}_0^\top {{\mathsf {A}}}_0{{\mathsf {x}}}= \sum _{i=1}^m [{{\mathsf {a}}}_i^\top {{\mathsf {x}}}]^2 \ge [ {{\mathsf {a}}}_j^\top {{\mathsf {x}}}]^2 = \varepsilon ^2 \Vert {{\mathsf {x}}}\Vert _1^2 >0\, . \end{aligned}$$
  Following this strategy, we can generate many constraints ${{\mathsf {B}}}_0 {{\mathsf {x}}}_0^{*} = {{\mathsf {b}}}_0$ by simply changing parameter $\varepsilon$, and ${{\mathsf {A}}}_0^\top {{\mathsf {A}}}_0$ is always strictly $\{{{\mathsf {x}}}\in {\mathbb {R}}^{n}_+ : {{\mathsf {B}}}_0 {{\mathsf {x}}}= {{\mathsf {o}}}\}$-copositive.
- **Choice 2**: We choose ${{\mathsf {B}}}_0$ with at least one row to be strictly positive, which means that the set ${{\mathsf {B}}}_0{{\mathsf {x}}}= {{\mathsf {b}}}_0$ is bounded and the recession cone of it shrinks to $\{{{\mathsf {o}}}\}$. By default, the chosen ${{\mathsf {B}}}_0$ satisfies the condition that ${{\mathsf {A}}}_0^\top {{\mathsf {A}}}_0$ is $\{{{\mathsf {x}}}\in {\mathbb {R}}^{n}_+ : {{\mathsf {B}}}_0 {{\mathsf {x}}}= {{\mathsf {o}}}\}$-copositive.

Following the construction, we then choose a suitable $\rho$ such that the constructed ${{\mathsf {x}}}_0^{*}$ is an optimal solution to the problem (17) or (18). To this end, we obtain a group of constrained soft sparse regression problems with known optimal solutions ${{\mathsf {x}}}_{0}^{*}$ and their objective value. After standardizing the constructed problems (subtracting a constant such that the optimal value is 0), by ${\mathfrak {S}}_{d,m,n}$ ($\bar{{\mathfrak {S}}}_{d,m,n}$) we denote problem sets that contain generated instances with randomly generated ${{\mathsf {A}}}_0 \in {\mathbb {R}}^{d \times n}$ and constructed ${{\mathsf {B}}}_0 \in {\mathbb {R}}^{m \times n}$ (randomly generated ${{\mathsf {B}}}_0 \in {\mathbb {R}}^{m \times n}$).

For hard sparse regression, an instance can be determined by the parameters ${{\mathsf {A}}}_0$, ${{\mathsf {a}}}_0$, ${{\mathsf {B}}}_0$, ${{\mathsf {b}}}_0$ and *k*. With same procedure adopted as in the soft case, we generate ${{\mathsf {A}}}_0$, ${{\mathsf {a}}}_0$, ${{\mathsf {B}}}_0$, ${{\mathsf {b}}}_0$ and a $k_0$-sparse vector ${{\mathsf {x}}}_{0}^{*}$. By taking $k = k_0$, a hard constrained sparse regression problem with known optimal solution ${{\mathsf {x}}}_{0}^{*}$ and optimal value is generated. Similarly, by ${\mathfrak {H}}_{d,m,n}$ ($\bar{{\mathfrak {H}}}_{d,m,n}$) we denote problem sets that contain generated instances with randomly generated ${{\mathsf {A}}}_0 \in {\mathbb {R}}^{d \times n}$ and constructed ${{\mathsf {B}}}_0 \in {\mathbb {R}}^{m \times n}$ (randomly generated ${{\mathsf {B}}}_0 \in {\mathbb {R}}^{m \times n}$).

#### Numerical results

We test our approach on a group of generated problem sets with each set containing 100 instances and record the dual bound as shown in Table 2. Recall that the optimal value of each constructed instance is 0. In Table 2, the label "$\tau$-regularized" indicates whether the regularization term $\tau \sum \limits _{i=1}^n (v_i^2+w_i^2)$ is applied for the problem set or not. Here and in the sequel, an entry $\approx 0$ signifies a number of magnitude $10^{-4}$ or smaller.

Overall, from Table 2, we can see that our approach provides an acceptable copositive dual bound from the perspective of average bound, ranging from $-0.015$ to $-0.15$. In some instances the best bound reported exceeds the optimal value, which may be a systematical error generated in the process of copositivity detection where we solve a MILP subproblem. In other words, some non-copositive matrices are considered copositive in very rare cases, which extends the feasible set of the dual problem. Recall that the Hessian of instances in problem sets ${\mathfrak {S}}_{d,m,n}$ or ${\mathfrak {H}}_{d,m,n}$ is strictly $L_\infty$-copositive by construction while the Hessian of instances in problem sets $\bar{{\mathfrak {S}}}_{d,m,n}$ or $\bar{{\mathfrak {H}}}_{d,m,n}$ may not be strictly $L_\infty$-copositive, which indicates that the strong conic duality may not hold for problems from $\bar{{\mathfrak {S}}}_{d,m,n}$ or $\bar{{\mathfrak {H}}}_{d,m,n}$. However, from Table 2, irrespective of whether the $\tau$-regularized strategy applied or not, it can be seen that the average bound of problems from $\bar{{\mathfrak {S}}}_{d,m,n}$ or $\bar{{\mathfrak {H}}}_{d,m,n}$ is still relatively tight in computational sense in general, even in absence of a strong duality guarantee. Moreover we note that our method performs better on hard regression instances, providing a conic dual bound with absolute gap less than 0.006 for hard instances. Regarding soft regression problems, across all problem sizes, our strategy gives a dual bound with an absolute gap of the order $10^{-2}$. While bounds generated by our approach are quite tight, the computational effort for it can be massive, in line with similar conic optimization models yielding results of comparable quality. When $n = 16$, all the instances from test sets ${\mathfrak {S}}_{d,m,n}(\bar{{\mathfrak {S}}}_{d,m,n})$ and ${\mathfrak {H}}_{d,m,n}(\bar{{\mathfrak {H}}}_{d,m,n})$ can be resolved within 5 minutes. As the problem dimensions increase to $n = 32$, the computational effort sees a dramatic growth, with 12% of instances needing longer than 10 minutes. In the worst case, runtime can be 20 minutes. For the cases $n = 64$, 73% instances are solved within 30 minutes while the worst case takes roughly 110 minutes.

**Table 2:** Soft sparse regression instances

Problem set	$\tau$-Regularized	Worst bound	Best bound	Average bound
${\mathfrak {S}}_{4,2,16}$	No	$-$ 0.13	0.00012	$-\,0.081$
$\bar{{\mathfrak {S}}}_{4,2,16}$	No	$-$ 0.18	$-\,0.0021$	$-\,0.089$
$\bar{{\mathfrak {S}}}_{4,2,16}$	Yes	$-$ 0.14	$-\,0.0026$	$-\,0.083$
${\mathfrak {S}}_{8,4,32}$	No	$-$ 0.22	$-\,0.0015$	$-\,0.091$
$\bar{{\mathfrak {S}}}_{8,4,32}$	No	$-$ 0.31	$-\,0.0032$	$-\,0.095$
$\bar{{\mathfrak {S}}}_{8,4,32}$	Yes	$-$ 0.24	$-\,0.0027$	$-\,0.092$
${\mathfrak {S}}_{16,6,64}$	No	$-$ 0.31	$-\,0.0021$	$-$ 0.11
$\bar{{\mathfrak {S}}}_{16,6,64}$	No	$-$ 0.42	$-0.0031$	$-$ 0.15
$\bar{{\mathfrak {S}}}_{16,6,64}$	Yes	$-$ 0.37	$-0.0037$	$-$ 0.12
${\mathfrak {H}}_{4,2,16}$	No	$-\,0.046$	0.00041	$-\,0.0015$
$\bar{{\mathfrak {H}}}_{4,2,16}$	No	$-\,0.061$	$\approx 0$	$-\,0.0021$
$\bar{{\mathfrak {H}}}_{4,2,16}$	Yes	$-\,0.054$	$\approx 0$	$-\,0.0018$
${\mathfrak {H}}_{8,4,32}$	No	$-\,0.062$	$\approx 0$	$-\,0.0019$
$\bar{{\mathfrak {H}}}_{8,4,32}$	No	$-\,0.065$	$\approx 0$	$-\,0.0042$
$\bar{{\mathfrak {H}}}_{8,4,32}$	Yes	$-\,0.063$	$\approx 0$	$-\,0.0037$
${\mathfrak {H}}_{16,6,64}$	No	$-\,0.067$	$\approx 0$	$-\,0.0041$
$\bar{{\mathfrak {H}}}_{16,6,64}$	No	$-\,0.071$	$\approx 0$	$-\,0.0053$
$\bar{{\mathfrak {H}}}_{16,6,64}$	Yes	$-\,0.070$	$\approx 0$	$-\,0.0047$

### Comparisons for soft sparse regression

In this subsection, we compare our method with four popular approximate approaches to soft sparse regression problems, from the perspective of optimal value.

For convenience of expression, we denote these alternatives by

$$\begin{aligned} R_{1}({{\mathsf {x}}}):=\Vert {{\mathsf {x}}}\Vert _{\frac{1}{2}}\, ,\quad R_{2}({{\mathsf {x}}}):=\Vert {{\mathsf {x}}}\Vert _{1}\, ,\quad R_{3}({{\mathsf {x}}}):=\Vert {{\mathsf {x}}}\Vert _{1}-\Vert {{\mathsf {x}}}\Vert _{2}\, . \end{aligned}$$

However, in general, the sparse solution obtained through these approximate sparse terms may differ from the one obtained via the original term $\Vert \cdot \Vert _{0}$. The exactness of these approximations holds only under some conditions. For example, it was proved in [17] that the sparse solution can be recovered exactly via $\Vert \cdot \Vert _1$ regularization term if the so-called *restricted isometry property (RIP)* condition holds. In particular, the non-convex terms $\Vert \cdot \Vert _{q}$ with $0<q<1$ can be regarded as a continuation strategy to approximate $\Vert \cdot \Vert _0$ as $q\searrow 0$, where the RIP is also needed for exactness. Similarly, a RIP-type sufficient condition is given in [37] to guarantee that with the regularization term $\Vert \cdot \Vert _{1}-\alpha \Vert \cdot \Vert _{2}$ added, a sparse vector can be recovered exactly.

**Fig. 1:**
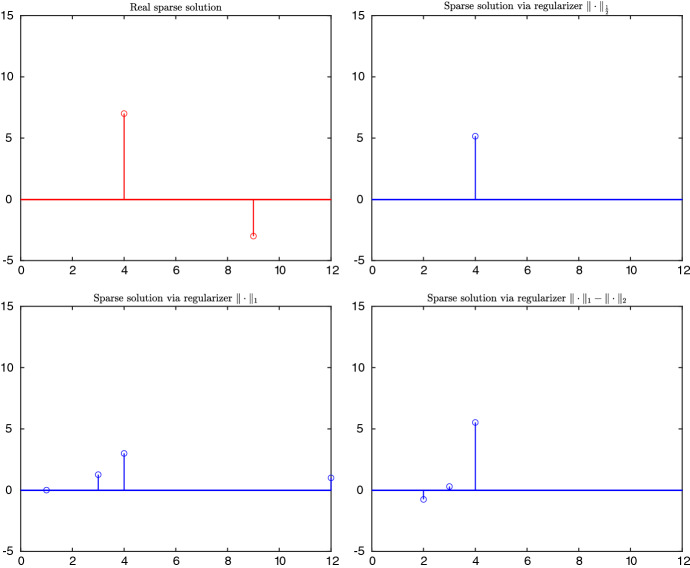
Row 1: original constructed solution and recovered sparse solution. Row 2: recovered sparse solutions

We illustrate this discrepancy in Fig. 1. In order to quantify this gap, we construct the following soft sparse regression example. With randomly generated matrix ${{\mathsf {A}}}_0\in {\mathbb {R}}^{3\times 12}$ and properly chosen sparse vector ${{\mathsf {x}}}_{0}^{*}\in {\mathbb {R}}^{12}$, we let ${{\mathsf {b}}}_0 := {{\mathsf {A}}}_0{{\mathsf {x}}}_{0}^{*}\in {\mathbb {R}}^3$ and choose $\rho =1$. Following the construction, ${{\mathsf {x}}}_0^{*}$ is the optimal solution to the problem

21 $$\begin{aligned} \min _{{{\mathsf {x}}}\in {\mathbb {R}}^{n}}F_0({{\mathsf {x}}}) := \frac{1}{2}\Vert {{\mathsf {A}}}_{0}{{\mathsf {x}}}-{{\mathsf {a}}}_{0}\Vert ^2+ 1 \cdot \Vert {{\mathsf {x}}}\Vert _0\, . \end{aligned}$$

By replacing the term $\Vert {{\mathsf {x}}}\Vert _0$ with sparse approximate regularization terms, we arrive at

22 $$\begin{aligned} \min _{{{\mathsf {x}}}\in {\mathbb {R}}^{n}}F_i({{\mathsf {x}}}) := \frac{1}{2}\Vert {{\mathsf {A}}}_{0}{{\mathsf {x}}}-{{\mathsf {a}}}_{0}\Vert ^2+1\cdot R_{i}({{\mathsf {x}}}), \quad i{ \in \! [{1}\! : \! {3}]} \, . \end{aligned}$$

By a heuristic algorithm [33], we obtain optimal solutions ${{\mathsf {x}}}_i^{*}$ for $F_i({{\mathsf {x}}})$, $i{ \in \! [{1}\! : \! {3}]}$, i.e. $F_i({{\mathsf {x}}}_i^{*}) = \min _{{{\mathsf {x}}}\in {\mathbb {R}}^{12}}F_i({{\mathsf {x}}})$. As presented in the Fig. 1, these solutions are distinct from the constructed real sparse solution ${{\mathsf {x}}}_0^*$.

In Table 3, the number in the *i*-th row and *j*-th column stands for the value of objective $F_i$ at the point ${{\mathsf {x}}}_{j}^{*}$. In addition, we highlight the best value of $F_i$ among all these points in bold. From the perspective of each row, the optimal solution ${{\mathsf {x}}}_i^{*}$ rather than original sparse solution minimizes the objective $F_i({{\mathsf {x}}})$, which means that the approximate models are not equivalent to the original sparse problem. In particular, we can see that, under original sparsity measure $\Vert \cdot \Vert _0$ (the last row), there exist considerable gaps between solutions obtained from approximate models and the original one.

**Table 3:** Objective values of different optimal solutions

Solution				
model	${{\mathsf {x}}}_1^{*}$	${{\mathsf {x}}}_2^{*}$	${{\mathsf {x}}}_3^{*}$	${{\mathsf {x}}}_0^{*}$
$F_1$	**7**.**4**	15.2	14.5	19.2
$F_2$	7.4	**5**.**6**	6.9	10.0
$F_3$	2.3	2.2	**1**.**3**	2.4
$F_0$	3.3	4.3	3.3	**2**.**0**

To further study the gap, we take problem sets ${\mathfrak {S}}_{d,m,n}$ as defined previously. Moreover, for the sake of convenient comparison, we let ${{\mathsf {B}}}_0 = {{\mathsf {O}}}$ and ${{\mathsf {b}}}_0 = {{\mathsf {o}}}$, which means that the problem set ${\mathfrak {S}}_{d,m,n}$ turns out to be ${\mathfrak {S}}_{d,0,n}$, unconstrained soft sparse regression problems of the form (21) with known optimal solution. We abbreviate it by ${\mathfrak {S}}_{d,n}$. On the other hand, we solve the approximate sparsity models (22) by algorithms that are frequently used in compressed sensing. In particular, when the regularization term is $\Vert \cdot \Vert _1$, we adopt the well-known fast iterative shrinkage-thresholding algorithm (FISTA), see [4]; when the regularization term is $\Vert \cdot \Vert _{\frac{1}{2}}$, we adopt half thresholding algorithm (HTA) from [38] which is a proximal algorithm for composite function with nonsmooth and nonconvex components; when the regularization term is $\Vert \cdot \Vert _{1} - \Vert \cdot \Vert _{2}$, we adopt the fast forward-backward splitting algorithm (FBS) from [34].

After running these algorithms for (22) on three problem sets with each set containing 100 instances, we obtain optimal solutions and have the Table 4. The numbers in Table 4 stand for the function values of $F_0({{\mathsf {x}}})$ at the points which are optimal solutions of the corresponding approximate sparsity models (22) solved by introduced algorithms. The number in the column with label "best" stands for the best case among all instances within a problem set while the "average" is the average performance. Recall that optimal values of the instances we constructed are 0. From Table 4, we can see that there is a large gap (exceeding 6) between the real optimal values and the results obtained by approximate models among the constructed instances, where the measurement matrix ${{\mathsf {A}}}_0$ may not satisfy RIP. However, shifting to Table 5, the conic dual bound we get from the same problem sets are still tight with absolute gap smaller than 0.23.

**Table 4:** Objective values of optimal solutions from approximate models

Problem sets	$F_1$			$F_2$			$F_3$		
	Best	Worst	Average	Best	Worst	Average	Best	Worst	Average
${\mathfrak {S}}_{4,16}$	4.0	9.0	6.3	7.0	14.0	10.2	3.0	14.0	10.9
${\mathfrak {S}}_{8,32}$	3.0	10.0	7.1	12.0	22.0	16.9	6.0	30.0	15.8
${\mathfrak {S}}_{16,64}$	6.0	12.0	8.7	19.0	28.0	22.8	10.0	19.0	13.9

**Table 5:** Conic dual bounds of the original models

Problem sets	$F_0$		
	Best	Worst	Average
${\mathfrak {S}}_{4,16}$	$-0.00013$	$-0.15$	$-0.082$
${\mathfrak {S}}_{8,32}$	$-0.00027$	$-0.21$	$-0.087$
${\mathfrak {S}}_{16,64}$	$-0.00018$	$-0.23$	$-0.085$

### Comparisons for hard sparse regression

In this subsection, we compare our method with 4 alternatives for hard sparse regression problems of the form (17) or (18) from the perspective of optimal value. As problem sets we take ${\mathfrak {H}}_{d,m,n}$ as defined previously, and each problem set contains 100 randomly generated instances. Moreover, the surrogates for sparsity constraint $\Vert {{\mathsf {x}}}\Vert _0\le k$ are:

- Top-(*k*,1) norm:
  $$\begin{aligned} \Vert {{\mathsf {x}}}\Vert _0\le k \quad \Leftrightarrow \quad \Vert {{\mathsf {x}}}\Vert _1 - \Vert |{{\mathsf {x}}}|\Vert _{k,1} = 0, \end{aligned}$$
  where $\Vert |x|\Vert _{k,1}$ denotes the $l_1$-norm of a subvector composed of top-*k* elements in absolute value;
- Top-(*k*,2) norm:
  $$\begin{aligned} \Vert {{\mathsf {x}}}\Vert _0\le k \quad \Leftrightarrow \quad \Vert {{\mathsf {x}}}\Vert _2^2 - \Vert |{{\mathsf {x}}}|\Vert _{k,2}^2 = 0, \end{aligned}$$
  where $\Vert |{{\mathsf {x}}}|\Vert _{k,2}$ denotes the $l_2$-norm of a subvector composed of top-*k* elements in absolute value;
- Big-M:
  $$\begin{aligned} \Vert {{\mathsf {x}}}\Vert _0\le k \quad \Leftrightarrow \quad |x_i|\le M u_i \, (i=1,2,\ldots ,n), \, {{\mathsf {e}}}^\top {{\mathsf {u}}}\le k, \, {{\mathsf {u}}}\in \{0,1\}^n, \end{aligned}$$
  where *M* is a sufficiently large constant;
- Big-M variant:
  $$\begin{aligned} \Vert {{\mathsf {x}}}\Vert _0\le k \quad \Leftrightarrow \quad |x_i|\le M u_i \, (i=1,2,\ldots ,n), \, {{\mathsf {e}}}^\top {{\mathsf {u}}}\le k, \, {{\mathsf {u}}}\in [0,1]^n,\, ({{\mathsf {e}}}-{{\mathsf {u}}})^\top {{\mathsf {u}}}\le 0\, . \end{aligned}$$

By replacing the sparsity constraint with these alternatives, we arrive at four problems. The first two we solve by PDCA [27], while for the others we employ the MIP solver Gurobi. Then we record corresponding optimal values as presented in Table 6 and compare with optimal dual bound generated by our method as shown in Table 7.

Table 6 evidences that, across all problem sets, the Big-M method provides best optimal values while the Big-M variant method performs worst. The possible reason for it might be the intractability of the involved complementarity constraint for Gurobi. However, the Big-M involved in the constraint is not easy to estimate if we do not know the optimal solutions. In contrast, our method is better than the other approaches without any a priori knowledge.

**Table 6:** Objective values of optimal solutions from approximate models

Problem sets	Methods	Best	Worst	Average
${\mathfrak {H}}_{4,2,16}$	Top-(*k*,1)	0.21	1.8	0.82
	Top-(*k*,2)	7.3	11.7	7.6
	Big-M	$\approx 0$	$\approx 0$	$\approx 0$
	Big-M variant	72.1	9.2	18.1
${\mathfrak {H}}_{8,4,32}$	Top-(*k*,1)	0.23	2.0	0.93
	Top-(*k*,2)	6.9	12.8	7.7
	Big-M	$\approx 0$	$\approx 0$	$\approx 0$
	Big-M variant	74.1	8.4	19.2
${\mathfrak {H}}_{16,6,64}$	Top-(*k*,1)	0.19	2.1	0.87
	Top-(*k*,2)	7.6	12.1	7.8
	Big-M	$\approx 0$	$\approx 0$	$\approx 0$
	Big-M variant	74.6	8.9	19.3

**Table 7:** Conic dual bounds of the original models

Problem sets	$F_h$		
	Best	Worst	Average
${\mathfrak {H}}_{4,2,16}$	0.00011	$-0.043$	$-0.0017$
${\mathfrak {H}}_{8,4,32}$	$\approx 0$	$-0.058$	$-0.0042$
${\mathfrak {H}}_{16,6,64}$	$\approx 0$	$-0.063$	$-0.0058$

## Conclusions and outlook

In this paper, we propose an exact completely copositive reformulation of QPCCs under mild conditions on the constraints, which extend previously known ones. An application on pursuing sparse solutions of (nonconvex) quadratic optimization is shown to satisfy our proposed conditions and therefore an equivalent completely positive optimization problem is obtained. Moreover, the condition for strong conic duality is also studied. To solve the conic optimization problem from the dual side, we introduce a direct search method with embedded MILP-based copositivity detection which does not need any large constants. Numerical experiments on sparse regression problems are reported which allow comparing our exact model with popular sparsity approximations, from the perspective of objective function values. However, the computational effort needed in our algorithm is relatively large because of the difficulty on the involved copositivity detection procedure for large problem sizes. A possible solution for it would be employing shortcut strategies in the copositivity checks which systematically exploit favourable instances by preprocessing [12].

## Data Availability

The code that supports the findings of this study are available upon request to the corresponding author.

## Footnotes

1 note﻿﻿ ﻿that we do *not* suggest to determine ${\mathfrak {C}}({{\mathsf {F}}})$ which may be exponential in size (as with the maximum-clique problem) but rather propose to exploit structural knowledge of the problem at hand; see Sect. 3, for instance.
